# Time-series metagenomic analysis reveals robustness of soil microbiome against chemical disturbance

**DOI:** 10.1093/dnares/dsv023

**Published:** 2015-10-01

**Authors:** Hiromi Kato, Hiroshi Mori, Fumito Maruyama, Atsushi Toyoda, Kenshiro Oshima, Ryo Endo, Genki Fuchu, Masatoshi Miyakoshi, Ayumi Dozono, Yoshiyuki Ohtsubo, Yuji Nagata, Masahira Hattori, Asao Fujiyama, Ken Kurokawa, Masataka Tsuda

**Affiliations:** 1 Graduate School of Life Sciences, Tohoku University, 2-1-1 Katahira, Sendai 980-8577, Japan; 2 Department of Biological Information, Graduate School of Bioscience and Biotechnology, Tokyo Institute of Technology, 2-12-1 Ookayama, Tokyo 152-8550, Japan; 3 Graduate School of Medicine, Kyoto University, Kyoto 606-8501, Japan; 4 Center for Information Biology, National Institute of Genetics, Mishima 411-8540, Japan; 5 Department of Computational Biology, Graduate School of Frontier Sciences, The University of Tokyo, 5-1-5 Kashiwanoha, Kashiwa 277-8561, Japan; 6 Principles of Informatics Research Division, National Institute of Informatics, Hitotsubashi, Tokyo 101-8430, Japan; 7 Earth-Life Science Institute, Tokyo Institute of Technology, 2-12-1 Ookayama, Tokyo 152-8550, Japan

**Keywords:** soil microbiome, metagenome, recalcitrant aromatic compounds, phage, robustness

## Abstract

Soil microbial communities have great potential for bioremediation of recalcitrant aromatic compounds. However, it is unclear which taxa and genes in the communities, and how they contribute to the bioremediation in the polluted soils. To get clues about this fundamental question here, time-course (up to 24 weeks) metagenomic analysis of microbial community in a closed soil microcosm artificially polluted with four aromatic compounds, including phenanthrene, was conducted to investigate the changes in the community structures and gene pools. The pollution led to drastic changes in the community structures and the gene sets for pollutant degradation. Complete degradation of phenanthrene was strongly suggested to occur by the syntrophic metabolism by *Mycobacterium* and the most proliferating genus, *Burkholderia*. The community structure at Week 24 (∼12 weeks after disappearance of the pollutants) returned to the structure similar to that before pollution. Our time-course metagenomic analysis of phage genes strongly suggested the involvement of the ‘kill-the-winner’ phenomenon (i.e. phage predation of *Burkholderia* cells) for the returning of the microbial community structure. The pollution resulted in a decrease in taxonomic diversity and a drastic increase in diversity of gene pools in the communities, showing the functional redundancy and robustness of the communities against chemical disturbance.

## 1. Introduction

Microbial communities in natural environments almost always encounter a wide variety of environmental changes (or ‘disturbances,’ in the field of ecology) in physical, chemical, and biological factors.^1^ It has been well recognized that indigenous microbial communities adapt to such changes through succession of their community structures.^2^ However, it still remains unclear (i) how the community *per se* responds to the change of environmental factors and (ii) which member(s) or group(s) and what kinds of genes in the community indeed play important roles in responding to the changes in each environment. Taking into consideration that >99% of environmental microbes are still unculturable under laboratory conditions, application of metagenomic approach has great advantages for investigating the structure and functional potentials of various microbial communities. Numerous ongoing projects involving deep sequencing of a variety of microbiota from different environments are providing a worldwide catalogue of metagenomic characteristics of microbial communities, and subsequent comparison of the characteristics is expected to clarify the environmental factors that govern the taxonomic and functional differences in the microbial communities.^3,4^ However, real-world environments are often subject to unpredictable and simultaneous changes of multiple physicochemical parameters in addition to frequent migration of organisms. For this reason, it is difficult to use metagenomic analysis of microbiota from natural environments to obtain information on the response of microbial community to the change of a specific environmental factor. Such information can be more definitively obtained by investigating the time-course change of microbiomes in a physically closed experimental ecosystem (i.e. microcosm) than by comparing a collection of ‘snapshots’ of metagenomes from various real-world microbial communities. Although several studies of time-course changes in human gut microbiomes have been reported,^5,6^ the application of such a strategy to soil environments is still challenging.^7,8^ This is because soil environments exhibit the most tremendous biodiversity on the Earth.^9–11^ Our aim of this study under the closed soil microcosm conditions was to investigate which members in a microbial community respond, and in what way, to the addition of chemical pollutants, which was considered as a change of one environmental factor. For this purpose, a closed soil microcosm was artificially amended simultaneously with four recalcitrant aromatic compounds, and the time-course changes in the taxonomic and gene compositions of the soil microbiota were investigated by metagenomic analysis. Our results revealed (i) drastic changes in the community structures and the gene sets for pollutant degradation, (ii) strong suggestion of the complete degradation of one aromatic compound by syntrophic metabolism with the involvement of two phylogenetically distant bacterial phyla, (iii) the return of community structure long after disappearance of the pollutants to the structure similar to that before pollution by the decrease of the most successfully propagated genus most probably through phage predation, and (iv) the redundancy and robustness of functional potentials of the community against the chemical disturbance.

## 2. Materials and methods

### 2.1 Preparation of artificially polluted soil samples

A farm soil (soil type: brown forest soil) at the Ehime Research Institute of Agriculture, Forestry, and Fisheries (33°58′42″ N and 132°48′03″ E), in Matsuyama, Japan^12^ was used in this study, and this soil had not been polluted with harmful aromatic compounds before our sampling. The soil was sampled at depths of 5–10 cm from the surface in April 2008, and large particles were removed with a 2-mm mesh sieve. The soil consisted of 77% sand, 11% of silt, and 12% clay, and was classified as sandy loam. Other physicochemical properties of the soil sample are summarized in Supplementary Table S1. The detailed procedure for artificially polluting the soil with four aromatic compounds, 3-chlorobenzoate (3CB), phenanthrene, biphenyl, and carbazole (i.e. 9-azafluerene; an aromatic heterocyclic organic compound), each at a final concentration of 125 mg/kg of wet soil, was described in our previous report.^12^ Each 200-g portion of the polluted or non-polluted control soil sample was transferred to a sterilized glass pot with a loose lid, and sterilized distilled water was then added to the soil to adjust its water content to 60% of the maximum water-holding capacity. The pots were incubated at 28°C in the dark for up to 24 weeks. Our monitoring of soil samples revealed the <8% changes in the water contents during the 24-week incubation (data not shown). At an appropriate time point after the incubation, all of the soil in each pot was fully harvested to (i) measure the amounts of pollutants, (ii) extract metagenomic DNA, (iii) store the microbial community at −80°C after its suspension in 15% glycerol or dimethyl sulfoxide solution, and (iv) store the remaining soil sample at −80°C.

### 2.2 Preparation of metagenomic DNA

Metagenomic DNA from 10-g soil sample was prepared by using a PowerMax^TM^ Soil DNA Isolation Kit (MO BIO Laboratories, Carlsbad, CA, USA). When the extracted DNA solution contained a large amount of humic substances, they were removed by repeating column purification step according to the protocol by the manufacture. The DNA solution from a total of 20-g soil was concentrated by ethanol precipitation, and used for the metagenomic sequencing and the templates for PCR reactions.

### 2.3 Pyrosequencing of 16S rRNA gene amplicons and their taxonomic analysis

The detailed analysis of the soil microbial communities on the basis of 16S rRNA gene sequences was performed by the Roche 454 pyrosequencing of PCR amplicons of 16S rRNA genes using 11 metagenomic DNA samples that were prepared from the control and polluted soils as well as the soil just before the pollution. Parts of the 16S rRNA genes [the V3–V4 region (positions from 342 to 806 in the *Escherichia coli* numbering)] were PCR-amplified using our non-degenerate universal primer set of 342F and 806R (Supplementary Table S2)^13^ and *EX Taq HS* polymerase (TAKARA BIO, Ohtsu, Japan). More detailed information on the primer set and PCR conditions has been described in our previous publication.^13^ After addition of the sequencing adapters A and B, the amplicons were sequenced using a Roche 454 GS FLX Titanium platform (Roche, Basel, Switzerland) according to the manufacturer's protocol.

Our previous protocol was also used to select high-quality 16S rRNA gene amplicon sequences (with >350 and <550 bases in lengths) and for their subsequent processing (i.e. alignment to a standard sense strand and removal of primer sequences for pyrosequencing). Sequence clustering of these high-quality reads was conducted using the UCLUST program^14^ version 6.0.307 with identity of ≥97% and query and reference coverage of ≥80%. Chimera clusters detected by the UCHIME program^15^ in *de novo* mode and its reference mode [with searching the reference Gold Database (DB) (http://drive5.com/uchime/gold.fa)] were removed. Taxonomic assignment of the resulting Roche 454 reads was performed by using the RDP Classifier program^16^ version 2.6 with bootstrap value ≥0.5 against the representative sequences of each 97% sequence cluster that was chosen by the UCLUST program. Unless otherwise stated, the programs for read assemblage and analysis in this study were run under default parameters.

### 2.4 Sequencing of metagenomic DNA using Illumina sequencer and subsequent filtering of low quality and eukaryotic and its viral DNA reads

The 11 soil metagenomic DNA samples (Supplementary Table S3) that were employed for the amplification of 16S rRNA genes and subsequent 454 pyrosequencing were also used for paired-end sequencing by an Illumina GA IIx platform (Illumina, San Diego, CA, USA). The metagenomic DNA was sheared to 200 bp (on average) by a Covaris-S instrument (Covaris, Woburn, MA, USA) to construct the DNA library, and its paired-end sequencing with a maximum read length of 75 bases (at the time of sequencing) was carried out using the base-calling pipeline versions 1.3 and 1.5.

The Illumina metagenomic reads that were evaluated as being of quality ‘*N*’ by the chastity filtering in the GA pipeline and those containing more than one nucleotide with the quality flag ‘B’ in the first 60 nucleotides were eliminated. The reads originated from eukaryotes and their viruses were identified as follows. The reads were searched by the BLASTX program^17^ version 2.2.26 with parameters ‘-e 50 -M PAM30’ against the GenBank-nr DB. The taxonomic origin of each read that fulfilled the parameters was next assigned by the highest BLAST scoring hit with identity ≥70% and BLAST bit-score ≥40 against the taxonomy of the database sequence in the NCBI Taxonomy DB (ftp://ftp.ncbi.nih.gov/pub/taxonomy/). The reads assigned to eukaryotic and eukaryotic viral origins were eliminated. The typical taxonomy nomenclature system is not applied to taxonomy of viruses, and their DNA sequences in the GenBank-nr DB are not associated with their taxonomic description. Therefore, use of only GenBank-nr DB was difficult to identify eukaryotic viral DNA reads in our metagenomic samples. To identify as many such reads as possible, our in-house eukaryotic viral protein sequence DB was constructed by manual removal of archaeal and bacterial virus sequences from the NCBI Virus genome DB (ftp://ftp.ncbi.nih.gov/genomes/Viruses/all.faa.tar.gz). The resulting in-house database was used for the BLASTX search with the same parameters and thresholds as used for the GenBank-nr DB analysis. When one of the paired-end reads was predicted to be eukaryotic or eukaryotic viral origin, the two reads constituting the paired-reads were eliminated.

### 2.5 Illumina read assembly and protein coding sequences prediction

Metagenomic reads from each sample were separately assembled using the IDBA-UD program^18^ version 1.1.0 with parameters ‘--mink 20 --maxk 70 --step 5’. The protein coding sequences (CDS) in the contigs were predicted by using the MetaGeneMark program^19^ version 2.10.

### 2.6 Taxonomic and functional assignment of Illumina metagenomic reads

For taxonomic assignment of the Illumina metagenomic reads, a nucleotide sequence similarity search was conducted using the BWA-MEM program^20^ version 0.7.10 with the strict parameters ‘-k 25 -w 10 -c 100000’ against the draft and complete prokaryote genome sequences in the NCBI DB in December 2013, and the draft sequences of the three *Burkholderia* strains and one *Mycobacterium* strain isolated from the polluted soil samples in this study.

Functional annotation of each read was performed by BLASTX search with parameters ‘-e 50 -M PAM30’ against the KEGG protein sequence DB^21^ that was obtained in February 2014. Taxonomic (phylum, class, order, family, and genus levels) and functional [KEGG Orthology (KO)-level] assignment was achieved for the reads that exhibited the highest BLAST scoring hit with identity ≥70% and BLAST bit-score ≥40. The KO abundance was calculated by dividing the number of reads belonging to the same KO by the median length of proteins that were annotated to the same KO in the KEGG protein DB. The KEGG pathway abundance was calculated by summing the abundances of KOs belonging to one pathway. To compare the abundances of individual KOs and their pathways among different samples, such abundances in each metagenomic sample were normalized by the number of genomes that were estimated as the average abundance of *gyrB* and 35 universal single-copy genes (USCGs),^22^ each of which is carried as a single-copy form by almost all prokaryotic genomes (Supplementary Table S4). (*gyrB* and USCGs are hereafter described as ‘*gyrB-*added USCGs’ in this paper.) This method for normalizing functional abundances among the samples was applied to all of the BLAST-based functional assignments in this study.

### 2.7 KEGG-based comparison of Illumina reads among different metagenomic samples

The time-course changes in the relative abundances of genes at their functional level in metagenomic samples from the control and polluted soils were compared by conducting *k*-means clustering of KEGG pathway abundances. The pathways that (i) are absent from prokaryotes, (ii) had a normalized number of maximum hits <0.0001 throughout the time course, and (iii) had zero abundance in the samples were not analysed. The variation in abundance of each KEGG pathway at an appropriate time point compared with the abundance at the previous time point, *V_p_*(*t*), was calculated as follows:

$$V_{p}(t)=\log_{2}\frac{K_{p}(t)}{K_{p}(t-1)},$$

where *K_p_*(*t*) is the abundance of pathway *p* at time point *t*, and *t* − 1 is the previous time point. Five *V_p_*(*t*) scores for each pathway were calculated for *t* = (1, 3, 6, 12, 24) and *t* − 1 = (0, 1, 3, 6, 12), and the five *V_p_*(*t*) scores for every pathway were used for conducting the *k*-means clustering. To infer the statistically optimal number of clusters for the separate pathways, we used the Calinski–Harabasz (CH) Index.^23,24^ This index considers the ratio between inter- and intra-cluster distances. A high value of the CH index suggests the robustness of the clustering by the cluster number. We chose a cluster number with the highest CH index. The *k*-means clustering and the calculation of the CH indexes were conducted using the R software version 3.1.1 (http://www.r-project.org/).

Overall differences in the taxonomic and functional gene compositions among the different samples were analysed by calculating the 1 – Pearson correlation coefficient of abundances of (i) 97% operational taxonomic unit (OTU), (ii) Genus, (iii) KO, and (iv) KEGG pathway among the samples, and were thereafter conducted through hierarchical clustering with the complete linkage algorithm by using the R software.

### 2.8 Comparison of gene pools among samples using all Illumina reads

To analyse overall differences in gene pools among the 11 metagenomic samples, all-against-all BLASTN (version 2.2.26) comparison for 121 pairs of samples (11 samples × 11 samples including self-comparisons) was conducted with parameters ‘-e 0.01’. The functional difference between each pair of samples was analysed by calculating the distance D2, which can normalize such differences by using the BLAST bit-scores of the best hit.^25,26^ After calculating the D2 values for all pairs of samples, the hierarchical clustering according to the D2 values with the complete linkage algorithm was conducted using the R software.

### 2.9 Construction of in-house protein sequence DB for aromatic compound-degrading enzymes and its use for functional assignment of Illumina metagenomic reads

The KEGG DB lacks a number of genes that have recently been deposited in public databases, and this is also the case with the genes involved in the upstream parts of microbial aerobic and anaerobic degradation routes for the pollutants. Another major inconvenience is that many enzymes for the aerobic degradation, especially those with dioxygenation activities, are encoded by three to four genes, and the substrate specificities of enzymes are determined mainly by the gene products for the terminal dioxygenase, but not by the remaining ones.^27^ Therefore, we constructed our own in-house DB consisting of amino acid sequences of the enzymes for the following aerobic and anaerobic degradation routes of 3CB, phenanthrene, biphenyl, and carbazole: phenanthrene-phthalate-protocatechuate route, biphenyl-benzoate route, carbazole-anthranilate route, naphthalene-salicylate route, catechol 1,2-and 2,3-dioxygenation route, gentisate route, anaerobic polycyclic aromatic hydrocarbon (naphthalene) route, and anaerobic benzoate and toluene route (see Supplementary Table S5 for the reaction steps in each route). Among the protein sequences available as the relevant enzyme names in the NCBI DB (in January 2014), the sequences accompanied by one or more publications about the bacterial degradation of aromatic compounds were first collected as the initial reference sequences of our in-house DB. Protein sequences in two other in-house DBs for the enzymes for degradation of aromatic compounds that were constructed by Vilchez-Vargas et al.^28^ and Guazzaroni et al.,^29^ and the protein sequences deposited with relevant KOs in the KEGG DB were also integrated into our DB. The reference sequence collection thus constructed was BLASTP-searched (version 2.2.26) with parameters ‘-e 1e-8 -b 10 -v 10’ and threshold identity ≥90%, query and reference sequence coverage ≥70% against the GenBank-nr DB in April 2014. The protein sequences that fulfilled these stringent parameters were incorporated into our final in-house DB (Supplementary Table S6). For the multisubunit enzymes, only the subunits with clearly determined substrate specificities were usually deposited in our database.

All the high-quality Illumina metagenomic reads were BLASTX-searched with parameters ‘-e 50 -M PAM30’ against our in-house DB, and the reads with identity ≥70% and BLAST bit-score ≥40 in the BLASTX results were collected as candidate-hit reads. These reads were next subjected to BLASTX search against the GenBank-nr DB with parameters ‘-e 50 -M PAM30’, and the reads that were the best hits to the sequences in the final in-house DB were further used for their functional assignment. The abundance of each gene for an aromatic compound-degrading enzyme in a metagenomic sample was normalized by (i) the amino acid length of the enzyme and (ii) the average abundance of the *gyrB*-added USCGs.

### 2.10 Analysis of phage genes and phage genome-derived contigs

Illumina metagenomic reads putatively from phage origins were surveyed using the BLASTX search with parameters ‘-e 50 -M PAM30’ against the ACLAME (A CLAssification of Mobile genetic Elements) phage protein sequence DB^30^ version 0.4. The abundance of each phage gene in each metagenomic sample was normalized by (i) the length of the phage gene, and (ii) the average abundances of the *gyrB*-added USCGs, which were calculated from the KEGG analysis.

All CDS found in all assembled contigs were BLASTP-searched with the parameter ‘-e 0.001’ against ‘Phage Orthologous Groups (POGs)’ DB^31^ to find out the contigs from phage genomes. All the Illumina reads that were allocated to such contigs were obtained using the Bowtie 2 program^32^ version 2.1.0. All such reads from the 11 metagenomic samples were assembled using the IDBA-UD program with parameters ‘--mink 20 --maxk 70 --step 5’ to construct more accurate phage gene-containing contigs. In this study, the ‘putative contigs from the phage genome’ were defined as those that (i) were >2 kb in length, (ii) belonged to the phage genome-related contigs, (iii) contained at least one CDS with an amino acid sequence identity ≥40% against proteins in the POGs DB, and (iv) contained at least one phage genome-derived region that could be identified using the PHAST (PHAge Search Tool) program.^33^ This program was also used to analyse their host taxa in which the phages can proliferate, and to evaluate the completeness of the phage genome. The abundance of the phage genome-derived contig DNA in each metagenomic sample was calculated by the numbers of hit reads counted by BLASTN search (100% identity and 100% query coverage) of Illumina metagenomic reads against the phage contigs. The abundance was normalized by the contig length and the numbers of *gyrB*-added USCGs.

Time-course change in abundance of the longest 44.5-kb phage genome-derived contig (Contig70-3) in metagenomic samples was investigated by long-range PCR amplification. Primer sets of PCR for scanning the phage genome were designed using the GenoFrag software^34^ version 2.1: the primer sets of 1–4 (1F-1R, 2R-2R, 3F-3R, and 4F-4R, respectively) for checking the size of the genome, and the primer set 5 (5F-5R) for checking the presence of circular or concatemer form of phage genome (Supplementary Table S2). The PCR reaction using PrimeSTAR GXL polymerase (TAKARA BIO) and the soil metagenomic DNA samples as the templates was performed under the condition of 30 cycles of 98°C for 10 s, 55°C for 15 s, and 68°C for 10 min. The sizes of the PCR products were determined by 0.8% agarose gel electrophoresis. A quantitative PCR of the phage genome was performed using the primer set 6, 6F-6R (Supplementary Table S2), SYBR Premix *Ex Taq* (TAKARA BIO), and a DNA engine OpticonTM2 system (MJ Research, Waltham, MA, USA).

## 3. Results and discussion

### 3.1 Overview and preliminary experiments

Accidental pollution of natural environments by harmful aromatic compounds has often occurred by a mixture of various compounds [e.g. various polycyclic aromatic hydrocarbons (PAHs) in petroleum and intermediate products, final products, and by-products from chemical plants], which range from biodegradative to highly persistent compounds. In order to investigate the microbial community-based degradation of such pollutants in natural environments as a model system, a farm soil sample from Ehime, Japan (see Section 2) was artificially polluted with a mixture of four aromatic compounds, 3CB (as a relatively biodegradable monocyclic and chlorinated compound) and carbazole, phenanthrene, and biphenyl (as much more recalcitrant compounds; a nitrogen-containing heterocyclic compound and PAHs, respectively), each at a concentration of ∼1 mM. Each 200-g portion of the polluted and control soil samples was transferred to a sterilized glass pot with a loose lid, generating many experimental pots. These pots were incubated at 28°C in the dark for up to 24 weeks. At an appropriate time point, all the soil harvested from each pot were used to investigate the decrease of pollutants and the number of culturable heterotrophs and to analyse the metagenome of soil microbiota. 3CB began to decrease immediately after the incubation and disappeared within the first 3 weeks. In contrast, carbazole and the two PAHs remained or decreased very slowly during these 3 weeks, then decreased rapidly and constantly, and disappeared at Week 12 (Supplementary Fig. S1). The numbers of colony-formable heterotrophs in the polluted soil samples during the 24-week incubation were similar with those in the control soil samples (Supplementary Fig. S2). On the basis of our preliminary survey of time-course changes in the taxonomic compositions of soil microbial communities in the pots (see Supplementary SN1), 11 soil metagenomic DNA samples were prepared at the time point just before the pollution (at Week 0) (designated 0 hereafter) and at five subsequent time points (at Weeks 1, 3, 6, 12, and 24) from the control (1C, 3C, 6C, 12C, and 24C) and polluted (1, 3, 6, 12, and 24 M) soils. These samples were used for (i) the PCR amplification of 16S rRNA gene fragments and their subsequent sequencing by the Roche 454 system to investigate the time-course changes in the taxonomic compositions, and (ii) paired-end metagenomic sequencing by the Illumina system to investigate the time-course changes in the functional and taxonomic compositions of gene pools.

### 3.2 Time-course taxonomic changes of microbial community members in soils

**Figure 1.**
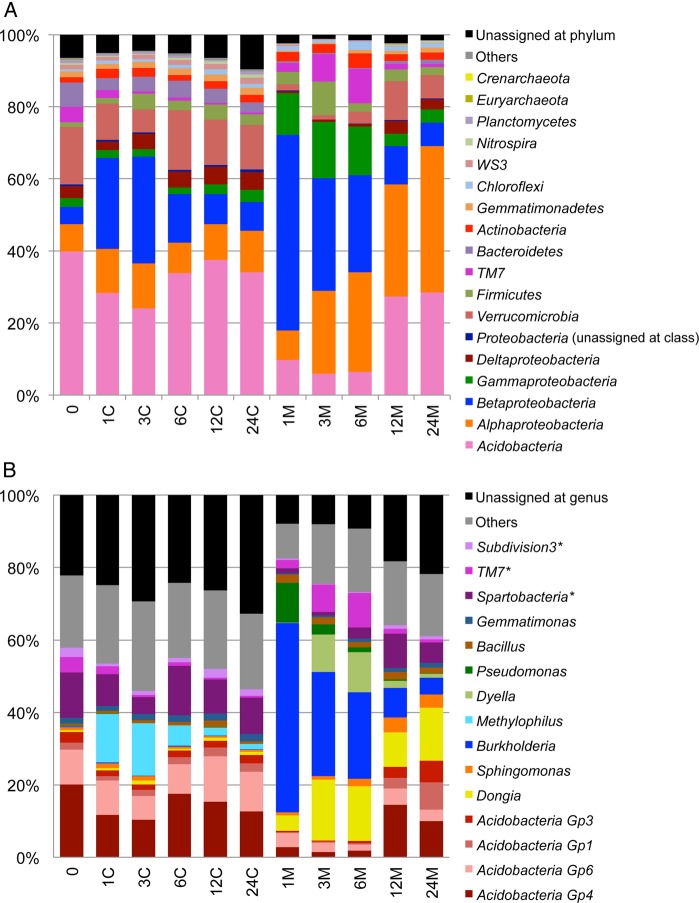
Phylum- and genus-level taxonomical succession of microbial communities in control and polluted soils. PCR amplicons of V3–V4 regions in the 16S rRNA genes from metagenomic samples were pyrosequenced, and taxonomically assigned by the RDP classifier (see the text for details). (A) Phylum-level succession. (B) Genus-level succession. C and M: metagenomic samples from the control and polluted soils, respectively. The numerals before C and M are the weeks after the pollution. Only the top 15 prokaryotic genera are shown for simplicity. Taxa with asterisks are genera *incertae sedis*.

Our pyrosequencing of PCR amplicons covering the V3–V4 regions of 16S rRNA genes from the 11 metagenomic samples yielded a total of 318,982 high-quality reads (average = 28,998 ± 3,311 per sample) (Supplementary Table S7). Use of the RDP Classifier led to the successful taxonomical assignment of 95.6 and 78.7% (on average) of the reads to phyla and genera, respectively, and the proportions of unassigned taxa at the phylum level were considerably low in the communities from the polluted soil at Weeks 1, 3, and 6 (Fig. 1A and Supplementary Table S7). The number of assigned genera in each sample was ∼250, and the proportion of *Archaea* in each of the 11 communities was <0.1%. The soil microbial community at Week 0 was, similarly to other reported soil microbial communities,^10^ dominated by the phyla *Acidobacteria* (especially subgroups *Gp4* and *Gp6*), *Verrucomicrobia* (especially genus *incertae sedis Spartobacteria*), and *Proteobacteria* (Fig. 1B). The community compositions in the control soil changed slightly during the 24-week incubation; the relative abundance of the genus *Methylophilus* (in *Proteobacteria*) increased at Weeks 1 and 3, and thereafter decreased. The increase in this methanol-utilizing genus was possibly due to a trace amount of residual methanol in the control soil; to pollute the soil uniformly with 3CB and the remaining very water-insoluble aromatic compounds, the four compounds were dissolved in methanol, which was thereafter removed as much as possible, and the same process without the pollutants was employed for preparation of the control soil (see Section 2). On the other hand, very drastic and complicated changes in the microbial community members, especially those in *Proteobacteria*, were observed in the polluted soil. Time-course changes in the relative abundance of the top 30 abundant genera (including genera *incertae sedis*) were further investigated by hierarchical clustering analysis (Supplementary Fig. S3), giving rise to four clusters (Clusters 1–4) on the basis of co-occurrence patterns. The genera that were dominant members at Week 0 but became minor ones immediately after the pollution belonged to Cluster 1, and only one-third of them (represented by *Acidobacteria Gp4* and *Spartobacteria*) regained their dominance after Week 12. The genera belonging to Cluster 2 (e.g. *Sphingomonas, Bradyrhizobium*, and subgroup *Gp1* in *Acidobacteria*) increased in their relative abundances after the disappearance of carbazole and the two PAHs. The remaining two clusters consisted of the genera that transiently increased in their relative abundances at Week 1 (e.g. *Burkholderia* and *Pseudomonas* in Cluster 4) and at Weeks 3 and 6 (e.g. *Dyella*, *Dongia*, *Mycobacterium*, *TM7*, and several anaerobes in Cluster 3). The genera in the last two clusters include various well-characterized aromatic compound-degrading strains,^35^ suggesting their direct or indirect involvement in the degradation of pollutants. The increase in relative abundances of 16S rRNA genes in the soil DNA samples did not definitively confirm the proliferation of their host cells in the soil environment. Since the three genera, *Burkholderia*, *Pseudomonas*, and *Mycobacterium*, were noteworthy taxa in the Illumina analysis of the metagenome (see below), the absolute abundances of their 16S rRNA genes in the control and polluted soil metagenomic samples were measured by quantitative PCR (Supplementary Fig. S4). The copy number of the 16S rRNA gene from each genus thus determined was in good agreement (Pearson correlations >0.90, *P*-value <0.01) with the relative abundance of the corresponding genus that was estimated by the assignment of 16S rRNA genes from the Roche 454 reads. This indicates that at least the three genera indeed proliferated in the polluted soil.

**Figure 2.**
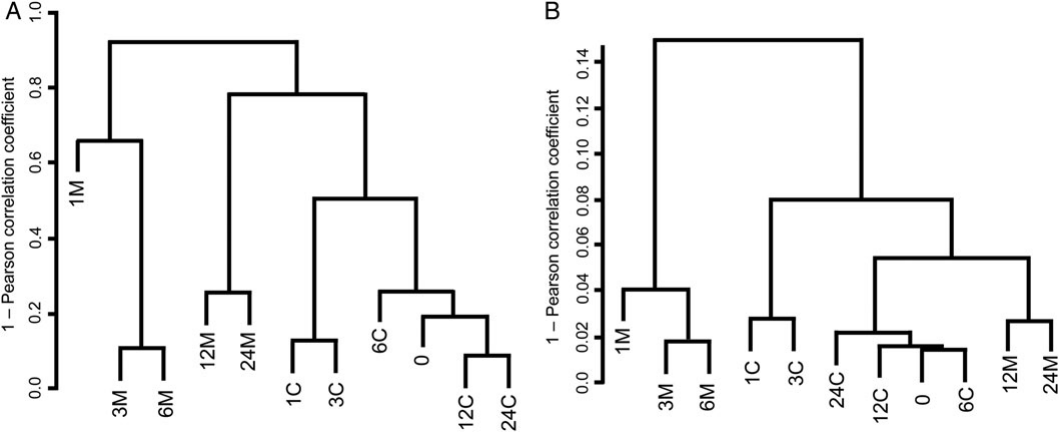
Hierarchical clustering analysis of taxonomic and functional compositions of 11 soil metagenomic samples. Clustering of (A) taxonomic compositions of 97% OTUs of 16S rRNA gene amplicons, and (B) functional compositions of genes for KEGG KO-assigned proteins was performed by comparison of the Euclidean distances. See the text for details.

Analysis of the changes in abundance of the top 30 genera throughout the 24-week time course did not conclusively reveal whether the microbial community in the polluted soil was still fluctuating at Week 24. However, our analysis of overall differences in the 16S rRNA gene compositions (at the 97% OTU level) among the 11 samples revealed that the community compositions of the polluted soil at Weeks 12 and 24 were (i) more similar to that at Week 0 than those at Weeks 1, 3, and 6, but (ii) still different from that at Week 0 (Fig. 2A). This implies that the microbial taxonomic compositions in the polluted soil tended to slowly, but not completely, return to that at Week 0. Such a gradual mode of return was more clearly observed in the case of the control soil. The resilience of microbial community compositions after environmental changes has been observed ubiquitously in various other environments, and the return to original compositions sometimes requires much longer periods (e.g. more than several years).^1,2,36^

### 3.3 Time-course changes of microbial gene pools in soils

The 11 soil DNA samples used for the pyrosequencing of 16S rRNA genes were also used for the metagenomic sequencing by the Illumina platform with a maximum read length of 75 bases (at the time of sequencing). The total number of reads from the 11 samples was 413 million (Supplementary Table S8). The total number of high-quality reads with >60 bases in lengths was ∼125 million (ranging from 7.3- to 14.4-million reads in each of the 11 samples), generating ∼9.3 Gb of sequences. Taxonomic and functional assignments of the Illumina-based metagenomic sequences in most other studies have been carried out after the assembly of reads.^21,37^ Therefore, our Illumina reads were assembled using the IDBA-UD software^18^ in order to obtain the contigs. The contig N50 median lengths from the 11 samples were very short (331–683 bases) (Supplementary Fig. S5), and only 205 and 9 contigs of >5 kb in length were obtained from the polluted and control soil samples, respectively. Our CDS prediction by the MetaGeneMark program generated a total of 3,333 CDS from the 214 contigs, and the subsequent BLASTP search against the GenBank-nr DB revealed that 3,095 CDS showed <50% identities (at the amino acid sequence level) with the functionally known genes or were assigned as hypothetical CDS (Supplementary Table S9). These results were consistent with the diverse taxonomic compositions of microbiota in our soil samples. Among the 63 CDS (32 and 31 CDS from polluted and control soils, respectively) that showed >70% identities (at the amino acid sequence level) with functionally annotated genes in the GenBank-nr DB, 15 CDS from the polluted soil samples were assigned to phage origins (see below for more details about the analysis of phage-inferred contigs), and the remaining 17 CDS were considered to have non-phage origins (e.g. genes for nucleotide metabolisms and ABC transporters). Most contigs from the control soil samples were probably the genome fragments derived from methylotrophs (Supplementary Table S9), and appeared not to encode the functions either directly or indirectly involved in the degradation of pollutants.

Because we obtained a huge number of very short contigs and only a small number of long contigs, the Illumina high-quality short reads were used for subsequent analysis. To evaluate the accuracy of the assignment of such short Illumina reads, a computer simulation experiment was conducted. The simulation of artificial 60-base ‘Illumina’ reads generated from the Sanger read data of a Minnesota soil microbiome^9^ revealed that identical assignments between the artificial Illumina data reads and their parental Sanger data reads at the phylum and KO levels were obtained with probabilities of 72 and 92%, respectively (Supplementary Table S10). These results reinforced the validity of our assignment of metagenomic sequences by using the Illumina short reads.

Taxonomic and functional assignment of the Illumina high-quality reads was performed by BLASTX search using the KEGG, GenBank-nr, and ACLAME DBs. The BLASTX search using the KEGG DB under the conditions of identity ≥70% and bit-score ≥40 led to the successful assignment of putative gene functions for 29% of the reads on average (ranging from 24 to 35%) (Supplementary Table S11 for gene abundance of each KO-assigned protein). Our analysis of overall differences in the relative abundances of genes for KO-assigned proteins among the 11 metagenomic DNA samples showed that the gene compositions at Weeks 1, 3, and 6 in the polluted soil samples formed a cluster very different from another cluster that was organized by the compositions from the remaining samples (Fig. 2B), indicating that the gene pools changed drastically in response to the chemical spike. Several metagenomic analyses of microbes in other real-world polluted soils also reported such changes in the gene pools.^29,38^ The gene compositions at Weeks 12 and 24 in our polluted samples were still different from that at Week 0 and those in the control samples. The overall relationship among the compositions of the genes for KO-assigned proteins (by the Illumina sequencing) in the 11 metagenomic samples was shown to have some similarity to the overall relationship among the compositions of 16S rRNA genes (by pyrosequencing) (Fig. 2). This implies that not only the taxonomic but also the gene compositions of the polluted soil microbiota tended to slowly return to those at Week 0. All the high-quality Illumina reads, including the functionally unassigned ones, were further compared among the 11 metagenomic DNA samples by the all-against-all BLASTN analysis, and subsequent clustering analysis exhibited that the gene compositions at Weeks 12 and 24 in the polluted soil were still distinct from that at Week 0 (Supplementary Fig. S6). The difference in the clustering patterns between all the reads and only the KO-assigned reads was likely ascribable to the biased property of KEGG DB, since this DB was constructed by using mainly the organisms for which complete genome sequences were available.

**Figure 3.**
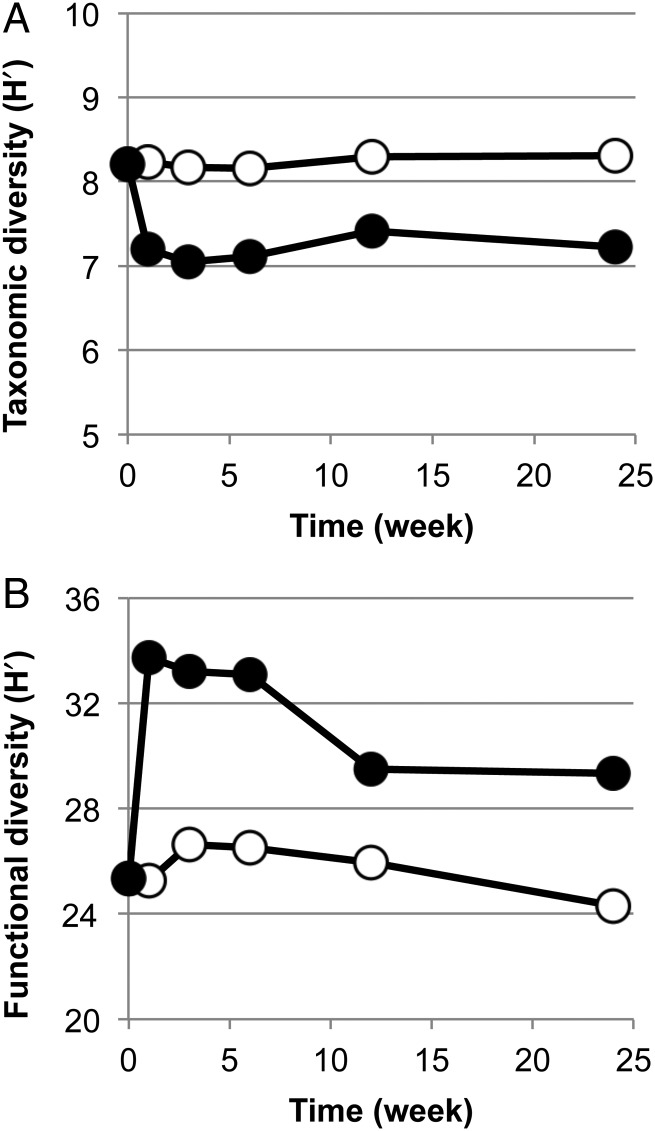
Time-course changes in taxonomic and functional diversity of metagenomes in control and polluted soils. (A) Taxonomic diversity. (B) Functional diversity. Open and closed circles: control and polluted soils, respectively. The Shannon-Wiener indices (H′) for taxonomic and functional diversities of the metagenomic samples were calculated from the compositions of 97% OTUs of the 16S rRNA gene amplicons and the compositions of genes for KO-assigned proteins, respectively.

The time-course changes in the taxonomic and functional diversities of metagenomic samples were investigated by the Shannon-Wiener indices. Although the pollution of soil led to a decrease in the diversity index of the taxonomic compositions of the microbial community (Fig. 3A), it also gave rise to a drastic increase in the diversity of the genes for KO-assigned proteins (Fig. 3B). This increase was most plausibly ascribable to the two most proliferating genera, *Burkholderia* and *Pseudomonas*, in the early weeks after the pollution. These bacteria have large genome sizes, and are well recognized to carry a number of genes apparently unique to these genera, especially for the catabolic genes for various aromatic compounds.^39–41^ A recent comparative metagenomic analysis of different microbiota from various tallgrass prairie soils indicated a positive correlation between the taxonomic and functional diversities of microbiomes.^42^ The difference between our closed soil microcosm samples and the real-world prairie soils may have been related to differences in environmental factors (chemical disturbance and physicochemical factors, respectively) or differences in the biological factors of the resident microbes.

In order to investigate what types of genes were enriched and correlated with one another during the drastic change in the gene compositions mentioned above, time-course variation patterns of KEGG pathway-level gene abundances (each of which was calculated by summing up the abundances of all genes for KO-assigned proteins in one pathway) were analysed by conducting *k*-means clustering. This analysis led to categorization of the pathway-level variation patterns into two clusters, designated M1 and M2 (Supplementary Table S12 and Fig. S7); while the pathway-level gene abundances for cluster M1 apparently remained constant during the 24-week incubation, those for cluster M2 oscillated drastically (a drastic increase at Week 1 followed by a rapid decrease at Week 3, and a slight re-increase at Week 6) and were well correlated with the degradation events of the pollutants. Of the 146 prokaryote-derived pathways (see Section 2), 100 pathways that include many core metabolic pathways were classified into cluster M1. The finding that there were no apparent variations in the abundance of genes for the core metabolic pathways in spite of the decrease in taxonomic diversity (Fig. 3A) might have been due to the functional redundancy of the genes for these common core pathways^2,43,44^ that were shared by the soil microbial members at Week 0 and the members at the weeks after the pollution. These results are in good agreement with other metagenomic studies that have addressed the degree of functional redundancy among the different human-associated microbial community compositions.^3^ The remaining 46 pathways belonged to cluster M2, which contains most of the degradation pathways of aromatic compounds (e.g. benzoate) as well as the pathways for the catabolism of aromatic amino acids and cell surface-related functions (i.e. cell motility, ABC-type transporters, and various secretion systems). The apparently synchronous variation patterns between the genes for the pollutant-degradation pathways and those for other functional groups (e.g. cell surface-related functions) in cluster M2 in the polluted soil might have been due to the genome structures of pollutant-degrading prokaryotes (and non-degrading and co-resident prokaryotes) that also possess the genes for the latter groups of pathways. In the control soil, the abundance of the genes that were assigned to the M2 cluster in the polluted soil oscillated slightly with the different patterns (Supplementary Table S13 and Fig. S7).

### 3.4 Time-course changes of microbial pollutant-degrading genes in soils

**Figure 4.**
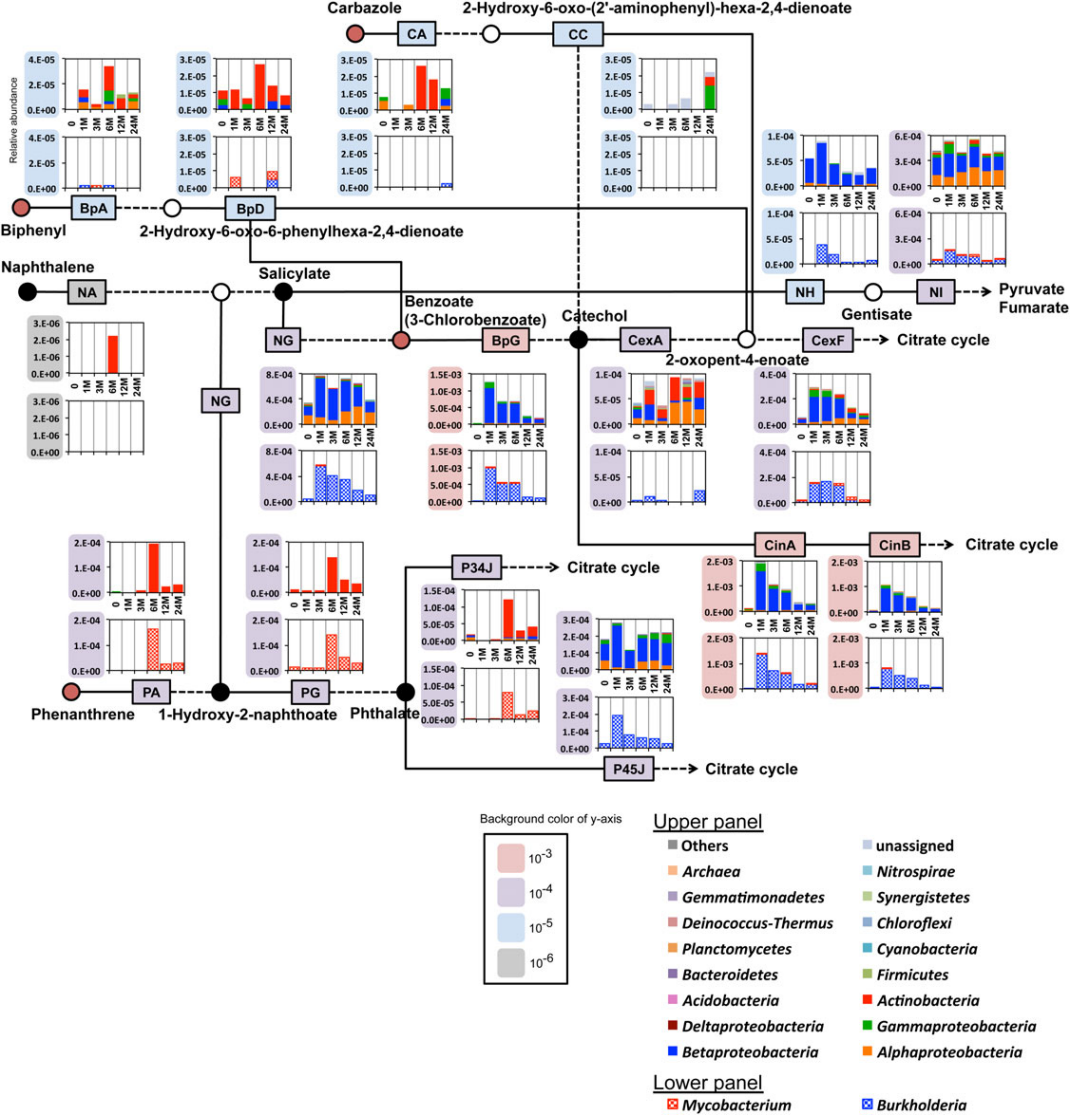
Time-course changes in abundances of genes for aerobic degradation of aromatic compounds in polluted soil. A simplified pathway map for well-known aerobic degradation routes of the four polluted compounds. Boxes located in each route indicate representative reaction steps, and several steps (dashed lines) are omitted for simplicity. Abbreviations of enzymes are shown in Supplementary Table S5. The two graphs indicate the taxonomic compositions and the abundance of the genes in each reaction step. The upper graph shows the abundances at the phylum level (with the exception that the domain and class levels are shown for *Archaea* and *Proteobacteria*, respectively), and the lower graph the abundances at the level limited to the genera of *Mycobacterium* and *Burkholderia*. See Section 2 for the details to calculate gene abundances. Depending on the abundances, the scales of *Y*-axes of graphs are conventionally categorized into four groups with the following colours: grey, 10^−6^; blue, 10^−5^; purple, 10^−4^; and red, 10^−3^. More detailed results at the overall pathway map level by using the metagenomic samples from the control and polluted soils are depicted in Supplementary Figs S8 and S9.

The change in the gene pools directly involved in the degradation of the pollutants was investigated using our in-house DB, which includes many amino acid sequences for the enzymes deposited recently in public DBs and those catalyzing aerobic and anaerobic degradation of the pollutants (see Section 2 and Supplementary Table S5). Our BLAST search of the high-quality Illumina reads against the in-house and GenBank-nr DBs (see Section 2) revealed that 66,433 reads most plausibly carried parts of genes for the degradation of pollutants. These reads were used to calculate the abundances and phylum-level taxonomic origins of the genes for the degradation reaction steps (Fig. 4; see also Supplementary Figs S8 and S9 for more comprehensive data). Our computer simulation of artificial ‘Illumina’ reads from the soil microbiome mentioned above indicated their ambiguous annotation of the short metagenomic reads at the genus level; the assignment accuracy was 34% for these reads. To clarify more accurately the genus-level taxonomic origins of the 66,433 metagenomic reads, nucleotide sequence similarity search was conducted for these reads using complete and draft prokaryote genome sequences (from 2,719 and 6,974 strains, respectively) in the NCBI DB and the draft sequences of three *Burkholderia* strains and one *Mycobacterium* strain that were isolated in this study from the polluted soil (see Supplementary SN2). This analysis revealed that the metagenomic reads whose origins were assigned to *Burkholderia* and *Mycobacterium* in the in-house DB exhibited the same taxonomic assignment with probabilities of 72 and 69%, respectively (Supplementary Table S14), indicating appropriate taxonomic assignments of the metagenomic reads at least for these two genera. The drastic increase in the abundance of *Burkholderia*-type genes for benzoate 1,2-dioxygenases (BpG; see Supplementary Table S5 for abbreviations of enzymes) (Fig. 4), which have also been indicated to convert 3CB to chlorinated catechol,^45^ coincided well with the relative abundance of *Burkholderia* cells (on the basis of community analysis by 16S rRNA gene profiling) as well as the rapid decrease of 3CB at Week 1. These data strongly suggested an important role of *Burkholderia*-type benzoate 1,2-dioxygenases in the degradation of 3CB. This suggestion was further supported by our preliminary transplantation experiment, in which the polluted soil-derived microbial population was inoculated into the sterilized soil; there was a rapid and drastic increase of *Burkholderia* after the pollution of the sterilized soil amended with 3CB (our unpublished observation). Another study on 3CB-amended soil also reported its degradation mainly by *Burkholderia*.^46^ It is noteworthy that the drastic increase of *Burkholderia*-type genes was observed in other reaction steps, such as phthalate 4,5-dioxygenation (P45J) (Fig. 4) and salicylate 1-hydroxylation (NG) at Week 1, at which time point no rapid degradation of the three other pollutants was observed. This simultaneous enrichment of degradation genes was most likely due to a common genomic feature of the genus *Burkholderia*; many of *Burkholderia* strains so far investigated encode the enzymes for the phthalate degradation route by its 4,5-dioxygenation (P45J) and for NG (note that salicylate and 1-hydroxy-2-naphthoate can be catabolized by the same enzyme^39,47,48^). The taxonomic compositions of aerobic degradation genes in the polluted soil at Week 6 indicated that an actinobacterial genus, *Mycobacterium*, was the most plausible contributor to the initial dioxygenation step of phenanthrene (PA) and the subsequent degradation to protocatechaute through the 3,4-dioxygenation sub-route of phthalate (P34J to P34L)^49^ (Fig. 4 and Supplementary Fig. S9), although the highest abundance of this genus in the communities of the polluted soil was 1% (on the basis of the16S rRNA gene profiling) at Week 6. The 4,5-dioxygenation sub-route from phthalate and a branching sub-route from 1-hydroxy-2-naphthoate (NG) were suggested to be mediated by the involvement of *Proteobacteria*, especially *Betaproteobacteria*. The genes for these sub-routes apparently showed biphasic response patterns in their abundances (increases at Weeks 1 and 6). Taking all of these observations into account, it is likely that the intermediate metabolites (e.g. phthalate and 1-hydroxy-2-naphthoate) of the mycobacterial degradation of phenanthrene in the soil were released to the extracellular environment^50^ and utilized by a variety of co-resident microbial groups. There are indeed several reports of syntrophic associations between two phyla for the complete degradation of recalcitrant compounds.^51^ A *Mycobacterium* strain, EPa45, with the ability to degrade phenanthrene to TCA compounds, was isolated from the polluted soil at Week 15. Our genome sequencing of EPa45 suggested its phenanthrene degradation to protocatechaute^52^ via the same route as used for mycobacterial phenanthrene degradation that was proposed by the Illumina metagenomic read analysis. The numbers of metagenomic reads that hit the well-known genes for the initial dioxygenation enzymes for biphenyl (BpA) and carbazole (CA) were quite small (<16 hit reads at Week 6), and this was consistent with the undetectable level of such genes in the metagenomic samples by PCR analysis (Supplementary SN1). These results suggested the involvement of as-yet-unknown gene(s) and metabolic pathway(s) for the degradation of the two aromatic compounds. Involvement of anaerobic degradation pathways was also ambiguous in the functional analysis of the metagenomic reads.

### 3.5 Time-course change of bacteriophage genes

**Figure 5.**
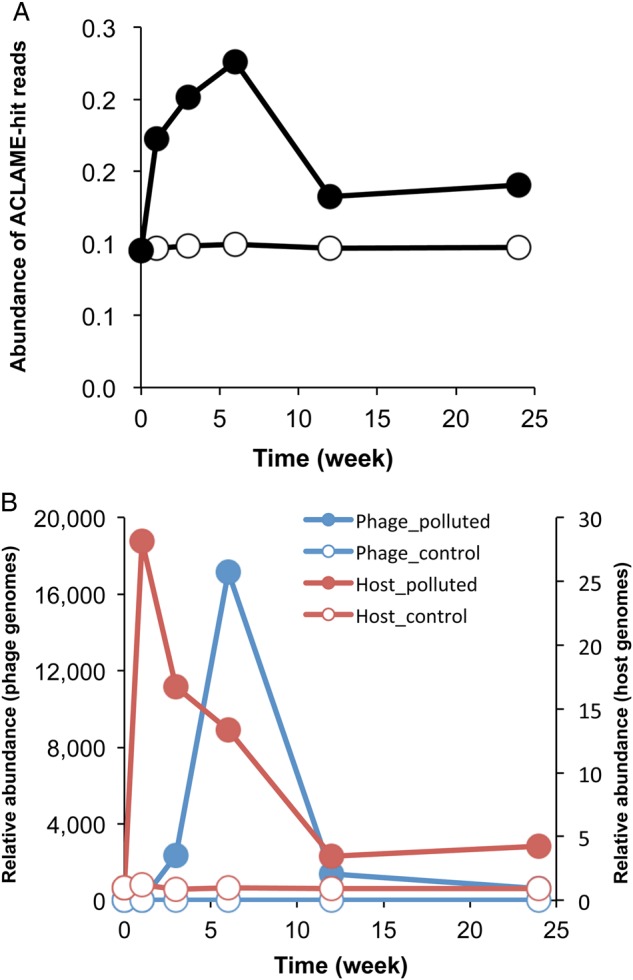
Time-course changes in abundances of phage genome-derived sequences in soil metagenomic samples. (A) Abundance of phage genome-derived reads in control (open circle) and polluted (closed) soil metagenomic samples based on BLASTX search of the Illumina reads against the ACLAME DB. (B) Relative abundances of a 44.5-kb contig (Contig70-3) DNA putatively derived from a phage genome from *Burkholderia* (blue circle) and the *Burkholderia* genomic DNA (red circle) in the control (open circle) and the polluted (closed circle) samples. The abundance of Contig70-3 DNA in each sample was based on the numbers of hit reads counted in BLASTN search of the metagenomic reads against Contig70-3, whereas the abundance of *Burkholderia* genomic DNA was based on the numbers of hit reads taxonomically assigned to *Burkholderia* by BLASTX search against the KEGG DB. The hit numbers in both cases were normalized by the number of *gyrB*-added USCGs in each metagenomic sample. The relative abundances of the Contig70-3 and *Burkholderia* genomes are expressed by taking the respective smallest values as 1.

Our BLASTX analysis of Illumina read-derived long contigs against the GenBank-nr DB suggested that some such contigs in the metagenomic samples from the polluted soil were originated from phage genomes (see above). More detailed BLASTX search of Illumina reads against the ACLAME DB indicated that the relative abundance of total phage DNA reads in the polluted soil increased at Weeks 3 and 6 (Fig. 5A), supporting the notion of an increase in prophage-carrying microbes and/or phage particles in the soil. Therefore, we further searched and analysed the phage gene-derived Illumina reads and their contigs (see Section 2.10). Among the re-assembled contigs from the Illumina metagenomic reads, 58 contigs had at least one CDS that showed >40% identity (at the amino acid sequence level) with phage CDS in the POGs DB (Supplementary Table S15), and these contigs were subjected to the PHAST analysis.^33^ This analysis revealed that (i) phage genome-derived regions were identified in eight large (15.6–93.6 kb) contigs, (ii) such contigs possessed many genes derived from *Pseudomonas* and *Burkholderia* phages, and (iii) a set of genes found in the 44.5-kb contig (Contig70-3) were evaluated as an ‘intact phage’ while the remaining contigs were evaluated as ‘incomplete phages’ (Supplementary Table S16). The BLASTN search of the Illumina reads from each of 11 metagenomic samples against the above-described 8 contigs was conducted to examine their quantitative time-course changes. This search showed an extensive increase of all 8 contigs at Week 3 or 6 only in the polluted soil (by 10^3^ to 10^4^ times greater than those at Week 0) and a subsequent decrease at Week 12, implying that replication of the phage genomes occurred around Weeks 3 and 6 (Fig. 5 and Supplementary Fig. S10). Our BLASTN search of all high-quality Illumina reads against the 8 contigs revealed that the Illumina hit reads were evenly distributed across the whole range of each contig, suggesting that the 8 contigs were not the chimeras that were artificially generated at our assemblage stage. Further analysis of Contig70-3 exhibited moderate (at most 45% at the BLASTX search level) similarity with the genome of a *Burkholderia* phage (Supplementary Table S16), BcepGomr (GI: 145321088), of soil origin.^53,54^ Our investigation of the relative abundances of the Illumina reads that corresponded to parts of Contig70-3 in each of 11 metagenomic samples indicated that such reads extensively increased only in the polluted soil (1.7 × 10^4^-fold increase at Week 6 compared with Week 1) (Fig. 5B). It is noteworthy that the relative abundance of the genomic DNA of *Burkholderia* in the polluted soil was the greatest at Week 1 (23-fold higher at this week than at Week 0). This result shows that there was temporal and numerical asynchrony between the relative abundances of the phage genome contig and its putative host genome. The metagenomic DNA sample at Week 6 from the polluted soil was used to PCR-amplify various Contig70-3-derived phage genome fragments, and we were able to detect (i) the amplicons of the phage-derived fragments of expected sizes and (ii) the phage genome structure with circular or concatemer form (Supplementary Fig. S11c). Quantitative PCR also confirmed the extensive increase of the phage genome at Week 6 in the polluted soil (Supplementary Fig. S11d). One plausible reason for the asynchrony is phage predation of *Burkholderia* cells around Week 6. Analysis of other long contigs also suggested that a similar situation was the case between *Pseudomonas* and its putative phages, although *in silico* analysis suggested that the phage-inferred contigs were not long enough to ‘intact’ phage genomes. Phage predation of successfully proliferating microbes, known as the ‘kill-the-winner’ hypothesis, has been proposed as one of the mechanisms underlying the compositional stability of microbial communities, and the involvement of this mechanism has been supported in aquatic environments.^55^ Our present findings strongly suggested that phage predation targeting the most successfully proliferating microbe (*Burkholderia* in this case) also contributed to the stabilization of our soil microbial community structure. It is unclear at present (i) what molecular mechanism(s) are involved in this phenomenon and (ii) whether such phages have lysogenic or lytic lifestyles.

### 3.6 Conclusions and perspectives

This study was performed to investigate by metagenomic approach the time-course changes in microbial taxonomic compositions and gene pools in the closed soil microcosm to which four aromatic compounds were simultaneously added. This approach strongly suggested the biphasic degradation of 3CB and phenanthrene: immediate degradation of the former compound mainly by *Burkholderia* and the delayed degradation of the latter one syntrophically by two phyla. Such suggestion was further supported by our traditional and molecular microbiological analysis as well as genomic analysis of relevant strains isolated from the polluted soil.

Systems biology has proposed that the robustness of biological systems at various levels is achieved by feedback control, fail-safe by means of diversity, and modularity.^43^ This proposal could be apparently applied to the microbiota in our closed soil microcosm: i.e. phage-mediated decrease of the most successfully propagated *Burkholderia* cells, functional redundancy of microbiota, and syntrophic degradation of pollutants, respectively. Although the proposal described above has been in part applicable to a few microbial communities such as anaerobic aquatic microbiota,^56^ it has not yet been investigated whether this is the case with soil microbiota. Our time-series metagenomic analysis of the closed soil microcosm polluted with aromatic compounds contributes to clarifying robustness of the soil microbial community against the chemical spike.

There existed over 250 genera in the soil at Week 0. Although some genera in the polluted soil showed drastic time-course changes in their relative abundances, most other genera only exhibited a ‘slight’ response to the pollution with no apparent increase or decrease. The ecological function of such ‘silent audiences’ in our microbial community remains to be explored. It is still unclear whether or not the increases in the degradation genes in our soil were followed by their expression for the degradation of pollutants. Time-course analysis of microbial communities using a combination of metagenomics with metatranscriptomics and metaproteomics, as well as other analyses (e.g. use of the stable isotope probing technique^29,57^), will greatly contribute to evaluation of the ecological functions of microbial genes at the community level.

## 4. Data availability

The nucleotide sequences determined in this study have been deposited in DDBJ/EBI/NCBI databases. The accession numbers for Sanger sequencing of clone library of 16S rRNA genes are LC016889–LC017690. The genome sequence of *Mycobacterium* strain is under the accession number of CP011773, and those of three *Burkholderia* strains are under the accession numbers of BBSL01000001–BBSL01000774, BBSM01000001–BBSM01000421, and BBSK01000001–BBSK01000186. All raw metagenome sequence data have been deposited under the Bioproject number of PRJDB2729 in the DDBJ Sequence Read Archive (Supplementary Table S3).

## Supplementary data

Supplementary data are available at www.dnaresearch.oxfordjournals.org.

## Funding

This work was supported by Grants-in-Aid for Scientific Research (no. 25292206 and Innovative Area ‘Genome Science’) from the Ministry of Education, Culture, Sports, Science and Technology, Japan, and by a grant from the Institute for Fermentation, Osaka (IFO) and a Global COE program ‘Ecosystem Adaptability Science for the Future’ at Tohoku University. Funding to pay the Open Access publication charges for this article was provided by the Ministry of Education, Culture, Sports, Science and Technology, Japan.

## Supplementary Material

Supplementary Data

## References

[1] Shade A., Peter H., Allison S.D. et al 2012, Fundamentals of microbial community resistance and resilience, Front. Microbiol., 3, 417. 23267351 10.3389/fmicb.2012.00417 PMC3525951

[2] Allison S.D., Martiny J.B. 2008, Resistance, resilience, and redundancy in microbial communities, Proc. Natl. Acad. Sci. USA, 105(Suppl. 1), 11512–19. 18695234 10.1073/pnas.0801925105 PMC2556421

[3] The Human Microbiome Project Consortium. 2012, Structure, function and diversity of the healthy human microbiome, Nature, 486, 207–14. 22699609 10.1038/nature11234 PMC3564958

[4] Gilbert J.A., Jansson J.K., Knight R. 2014, The Earth Microbiome project: successes and aspirations, BMC Biol., 12, 69. 25184604 10.1186/s12915-014-0069-1 PMC4141107

[5] Sharon I., Morowitz M.J., Thomas B.C., Costello E.K., Relman D.A., Banfield J.F. 2013, Time series community genomics analysis reveals rapid shifts in bacterial species, strains, and phage during infant gut colonization, Genome Res., 23, 111–20. 22936250 10.1101/gr.142315.112 PMC3530670

[6] Koenig J.E., Spor A., Scalfone N. et al 2011, Succession of microbial consortia in the developing infant gut microbiome, Proc. Natl. Acad. Sci. USA, 108(Suppl. 1), 4578–85. 20668239 10.1073/pnas.1000081107 PMC3063592

[7] Mackelprang R., Waldrop M.P., Deangelis K.M. et al 2011, Metagenomic analysis of a permafrost microbial community reveals a rapid response to thaw, Nature, 480, 368–71. 22056985 10.1038/nature10576

[8] Delmont T.O., Prestat E., Keegan K.P. et al 2012, Structure, fluctuation and magnitude of a natural grassland soil metagenome, ISME J., 6, 1677–87. 22297556 10.1038/ismej.2011.197 PMC3498926

[9] Tringe S.G., von Mering C., Kobayashi A. et al 2005, Comparative metagenomics of microbial communities, Science, 308, 554–7. 15845853 10.1126/science.1107851

[10] Janssen P.H. 2006, Identifying the dominant soil bacterial taxa in libraries of 16S rRNA and 16S rRNA genes, Appl. Environ. Microbiol., 72, 1719–28. 16517615 10.1128/AEM.72.3.1719-1728.2006 PMC1393246

[11] Luo C.W., Rodriguez-R L.M., Johnston E.R. et al 2014, Soil microbial community responses to a decade of warming as revealed by comparative metagenomics, Appl. Environ. Microbiol., 80, 1777–86. 24375144 10.1128/AEM.03712-13 PMC3957593

[12] Nagayama H., Sugawara T., Endo R. et al 2015, Isolation of oxygenase genes for indigo-forming activity from an artificially polluted soil metagenome by functional screening using *Pseudomonas putida* strains as hosts, Appl. Microbiol. Biotechnol., 99, 4453–70. 25573469 10.1007/s00253-014-6322-2

[13] Mori H., Maruyama F., Kato H. et al 2014, Design and experimental application of a novel non-degenerate universal primer set that amplifies prokaryotic 16S rRNA genes with a low possibility to amplify eukaryotic rRNA genes, DNA Res., 21, 217–27. 24277737 10.1093/dnares/dst052 PMC3989492

[14] Edgar R.C. 2010, Search and clustering orders of magnitude faster than BLAST, Bioinformatics, 26, 2460–1. 20709691 10.1093/bioinformatics/btq461

[15] Edgar R.C., Haas B.J., Clemente J.C., Quince C., Knight R. 2011, UCHIME improves sensitivity and speed of chimera detection, Bioinformatics, 27, 2194–200. 21700674 10.1093/bioinformatics/btr381 PMC3150044

[16] Wang Q., Garrity G.M., Tiedje J.M., Cole J.R. 2007, Naive Bayesian classifier for rapid assignment of rRNA sequences into the new bacterial taxonomy, Appl. Environ. Microbiol., 73, 5261–7. 17586664 10.1128/AEM.00062-07 PMC1950982

[17] Altschul S.F., Gish W., Miller W., Myers E.W., Lipman D.J. 1990, Basic local alignment search tool, J. Mol. Biol., 215, 403–10. 2231712 10.1016/S0022-2836(05)80360-2

[18] Peng Y., Leung H.C.M., Yiu S.M., Chin F.Y.L. 2012, IDBA-UD: a *de novo* assembler for single-cell and metagenomic sequencing data with highly uneven depth, Bioinformatics, 28, 1420–8. 22495754 10.1093/bioinformatics/bts174

[19] Zhu W.H., Lomsadze A., Borodovsky M. 2010, *Ab initio* gene identification in metagenomic sequences, Nucleic Acids Res., 38, e132. 20403810 10.1093/nar/gkq275 PMC2896542

[20] Li H., Durbin R. 2010, Fast and accurate long-read alignment with Burrows-Wheeler transform, Bioinformatics, 26, 589–95. 20080505 10.1093/bioinformatics/btp698 PMC2828108

[21] Qin J., Li R., Raes J. et al 2010, A human gut microbial gene catalogue established by metagenomic sequencing, Nature, 464, 59–65. 20203603 10.1038/nature08821 PMC3779803

[22] Raes J., Korbel J.O., Lercher M.J., von Mering C., Bork P. 2007, Prediction of effective genome size in metagenomic samples, Genome Biol., 8, R10. 17224063 10.1186/gb-2007-8-1-r10 PMC1839125

[23] Caliński T., Harabasz J. 1974, A dendrite method for cluster analysis, Commun. Stat., 3, 1–27.

[24] Arumugam M., Raes J., Pelletier E. et al 2011, Enterotypes of the human gut microbiome, Nature, 473, 174–80. 21508958 10.1038/nature09944 PMC3728647

[25] Korbel J.O., Snel B., Huynen M.A., Bork P. 2002, SHOT: a web server for the construction of genome phylogenies, Trends Genet., 18, 158–62. 11858840 10.1016/s0168-9525(01)02597-5

[26] Kurokawa K., Itoh T., Kuwahara T. et al 2007, Comparative metagenomics revealed commonly enriched gene sets in human gut microbiomes, DNA Res., 14, 169–81. 17916580 10.1093/dnares/dsm018 PMC2533590

[27] Parales R.E. 2003, The role of active-site residues in naphthalene dioxygenase, J. Ind. Microbiol. Biotechnol., 30, 271–8. 12695887 10.1007/s10295-003-0043-3

[28] Vilchez-Vargas R., Geffers R., Suarez-Diez M. et al 2013, Analysis of the microbial gene landscape and transcriptome for aromatic pollutants and alkane degradation using a novel internally calibrated microarray system, Environ. Microbiol., 15, 1016–39. 22515215 10.1111/j.1462-2920.2012.02752.x

[29] Guazzaroni M.E., Herbst F.A., Lores I. et al 2012, Metaproteogenomic insights beyond bacterial response to naphthalene exposure and bio-stimulation, ISME J., 7, 122–36. 22832345 10.1038/ismej.2012.82 PMC3526184

[30] Leplae R., Lima-Mendez G., Toussaint A. 2010, ACLAME: a classification of mobile genetic elements, update 2010, Nucleic Acids Res., 38, D57–61. 19933762 10.1093/nar/gkp938 PMC2808911

[31] Kristensen D.M., Waller A.S., Yamada T., Bork P., Mushegian A.R., Koonin E.V. 2013, Orthologous gene clusters and taxon signature genes for viruses of prokaryotes, J. Bacteriol., 195, 941–50. 23222723 10.1128/JB.01801-12 PMC3571318

[32] Langmead B., Salzberg S.L. 2012, Fast gapped-read alignment with Bowtie 2, Nat. Methods, 9, 357–9. 22388286 10.1038/nmeth.1923 PMC3322381

[33] Zhou Y., Liang Y., Lynch K.H., Dennis J.J., Wishart D.S. 2011, PHAST: a fast phage search tool, Nucleic Acids Res., 39, W347–52. 21672955 10.1093/nar/gkr485 PMC3125810

[34] Ben Zakour N., Gautier M., Andonov R. et al 2004, GenoFrag: software to design primers optimized for whole genome scanning by long-range PCR amplification, Nucleic Acids Res., 32, 17–24. 14704339 10.1093/nar/gkg928 PMC373259

[35] Peng R.H., Xiong A.S., Xue Y. et al 2008, Microbial biodegradation of polyaromatic hydrocarbons, FEMS Microbiol. Rev., 32, 927–55. 18662317 10.1111/j.1574-6976.2008.00127.x

[36] Hartmann M., Niklaus P.A., Zimmermann S. et al 2014, Resistance and resilience of the forest soil microbiome to logging-associated compaction, ISME J., 8, 226–44. 24030594 10.1038/ismej.2013.141 PMC3869018

[37] Oh S., Caro-Quintero A., Tsementzi D. et al 2011, Metagenomic insights into the evolution, function, and complexity of the planktonic microbial community of Lake Lanier, a temperate freshwater ecosystem, Appl. Environ. Microbiol., 77, 6000–11. 21764968 10.1128/AEM.00107-11 PMC3165412

[38] Yergeau E., Sanschagrin S., Beaumier D., Greer C.W. 2012, Metagenomic analysis of the bioremediation of diesel-contaminated Canadian high arctic soils, PLoS ONE, 7, e30058. 22253877 10.1371/journal.pone.0030058 PMC3256217

[39] Perez-Pantoja D., Donoso R., Agullo L. et al 2012, Genomic analysis of the potential for aromatic compounds biodegradation in *Burkholderiales*, Environ. Microbiol., 14, 1091–117. 22026719 10.1111/j.1462-2920.2011.02613.x

[40] Leekitcharoenphon P., Buzard G.S., Ussery D.W. 2014, Comparative genomics in the genus *Burkholderia*. In: Coenye T., Mahenthiralingam E. (eds), Burkholderia: From Genomes to Function. Caister Academic Press: Norfolk, UK, pp. 31–50.

[41] Silby M.W., Winstanley C., Godfrey S.A.C., Levy S.B., Jackson R.W. 2011, *Pseudomonas* genomes: diverse and adaptable, FEMS Microbiol. Rev., 35, 652–80. 21361996 10.1111/j.1574-6976.2011.00269.x

[42] Fierer N., Ladau J., Clemente J.C. et al 2013, Reconstructing the microbial diversity and function of pre-agricultural tallgrass prairie soils in the United States, Science, 342, 621–4. 24179225 10.1126/science.1243768

[43] Kitano H. 2004, Biological robustness, Nat. Rev. Genet., 5, 826–37. 15520792 10.1038/nrg1471

[44] Souza R.C., Hungria M., Cantao M.E., Vasconcelos A.T.R., Nogueira M.A., Vicente V.A. 2015, Metagenomic analysis reveals microbial functional redundancies and specificities in a soil under different tillage and crop-management regimes, Appl. Soil Ecol., 86, 106–12.

[45] Ogawa N., Miyashita K., Chakrabarty A.M. 2003, Microbial genes and enzymes in the degradation of chlorinated compounds, Chem. Rec., 3, 158–71. 12900936 10.1002/tcr.10059

[46] Morimoto S., Togami K., Ogawa N., Hasebe A., Fujii T. 2005, Analysis of a bacterial community in 3-chlorobenzoate-contaminated soil by PCR-DGGE targeting the 16S rRNA gene and benzoate 1,2-dioxygenase gene (*benA*), Microbes Environ., 20, 151–9.

[47] Chang H.K., Zylstra G.J. 1998, Novel organization of the genes for phthalate degradation from *Burkholderia cepacia* DBO1, J. Bacteriol., 180, 6529–37. 9851995 10.1128/jb.180.24.6529-6537.1998 PMC107754

[48] Balashova N.V., Stolz A., Knackmuss H.J., Kosheleva I.A., Naumov A.V., Boronin A.M. 2001, Purification and characterization of a salicylate hydroxylase involved in 1-hydroxy-2-naphthoic acid hydroxylation from the naphthalene and phenanthrene-degrading bacterial strain *Pseudomonas putida* BS202-P1, Biodegradation, 12, 179–88. 11826899 10.1023/a:1013126723719

[49] Stingley R.L., Khan A.A., Cerniglia C.E. 2004, Molecular characterization of a phenanthrene degradation pathway in *Mycobacterium vanbaalenii* PYR-1, Biochem. Biophys. Res. Commun., 322, 133–46. 15313184 10.1016/j.bbrc.2004.07.089

[50] Moody J.D., Freeman J.P., Doerge D.R., Cerniglia C.E. 2001, Degradation of phenanthrene and anthracene by cell suspensions of *Mycobacterium* sp. strain PYR-1, Appl. Environ. Microbiol., 67, 1476–83. 11282593 10.1128/AEM.67.4.1476-1483.2001 PMC92757

[51] Le Digabel Y., Demaneche S., Benoit Y., Vogel T.M., Fayolle-Guichard F. 2013, Ethyl *tert*-butyl ether (ETBE) biodegradation by a syntrophic association of *Rhodococcus* sp. IFP 2042 and *Bradyrhizobium* sp. IFP 2049 isolated from a polluted aquifer, Appl. Microbiol. Biotechnol., 97, 10531–39. 23474617 10.1007/s00253-013-4803-3

[52] Kato H., Ogawa N., Ohtsubo Y. et al 2015, Complete genome sequence of a phenanthrene degrader, *Mycobacterium* sp. strain EPa45 (NBRC 110737), isolated from a phenanthrene-degrading consortium, Genome Announc., 3, e00782-15. 10.1128/genomeA.00782-15 PMC4505128 26184940

[53] Summer E.J., Gill J.J., Upton C., Gonzalez C.F., Young R. 2007, Role of phages in the pathogenesis of *Burkholderia*, or 'where are the toxin genes in *Burkholderia* phages?’, Curr. Opin. Microbiol., 10, 410–7. 17719265 10.1016/j.mib.2007.05.016 PMC2064068

[54] Lynch K.H., Dennis J.J. 2014, Genomics of *Burkholderia* phages. In: Coenye T., Mahenthiralingam E. (eds), Burkholderia: From Genomes to Function. Caister Academic Press: Norfolk, UK, pp. 230–49.

[55] Rodriguez-Brito B., Li L., Wegley L. et al 2010, Viral and microbial community dynamics in four aquatic environments, ISME J., 4, 739–51. 20147985 10.1038/ismej.2010.1

[56] Stenuit B., Agathos S.N. 2015, Deciphering microbial community robustness through synthetic ecology and molecular systems synecology, Curr. Opin. Biotechnol., 33, 305–17. 25880923 10.1016/j.copbio.2015.03.012

[57] Muller E.E.L., Pinel N., Laczny C.C. et al 2014, Community-integrated omics links dominance of a microbial generalist to fine-tuned resource usage, Nat. Commun., 5, 5603. 25424998 10.1038/ncomms6603 PMC4263124

