# Supplementary material for: Time-series metagenomic analysis reveals robustness of soil microbiome against chemical disturbance
Source: DNA Res. 2015 Oct 1;22(6):413–24. doi: 10.1093/dnares/dsv023 (PMC4675710; doi:10.1093/dnares/dsv023)
Supplement: Supplementary Data [file supp_22_6_413__index.html]

Time-series metagenomic analysis reveals robustness of soil microbiome against chemical disturbance — Supplementary Data 

# Time-series metagenomic analysis reveals robustness of soil microbiome against chemical disturbance

## Supplementary Data

Supplementary Data

- Supplementary Figures - pdf file
- Supplementary Tables - xls file
- Supplementary Notes - pdf file
