# Supplementary material for: Time-series metagenomic analysis reveals robustness of soil microbiome against chemical disturbance
Source: DNA Res. 2015 Oct 1;22(6):413–24. doi: 10.1093/dnares/dsv023 (PMC4675710; doi:10.1093/dnares/dsv023)
Supplement: Supplementary Data [file supp_dsv023_dsv023supp_figures.pdf]

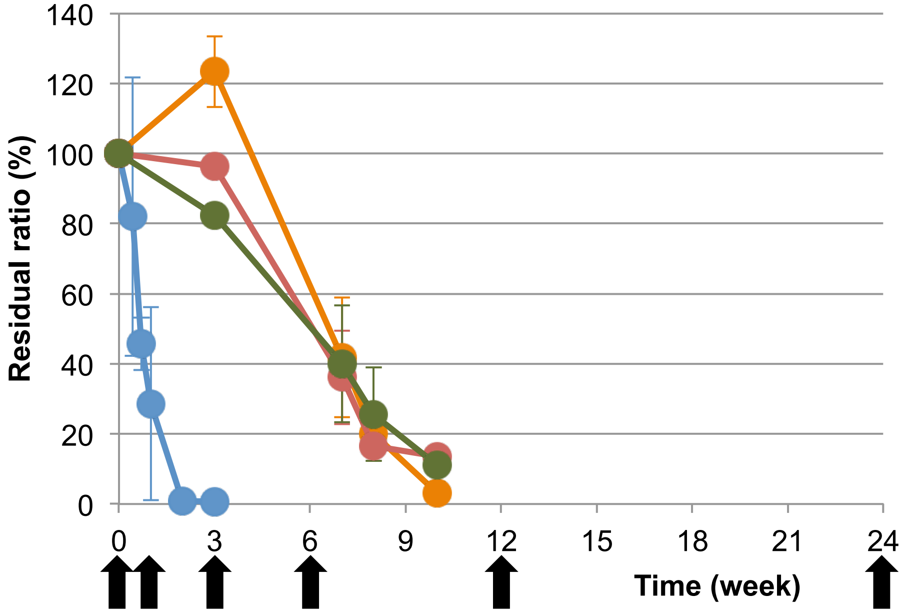

Supplementary Figure S1. Degradation of aromatic compounds in soil samples.
Symbols: blue circle, 3-chlorobenzoate; orange circle, biphenyl; red circle, phenanthrene; and green circle, carbazole. Time-course changes in the percentage values of residual to added chemical compound are depicted. To investigate the decrease of the pollutants in the soil samples, three-gram soil was suspended in 6 ml of hexane-acetone (1:1) solution, sonicated for 2 hours, and centrifuged for 5 min at 5,500 x *g*. The resulting extract was subjected to GC-MS analysis to measure the amounts of pollutants by the method described previously.[12] The measurement was performed using three independently extracted samples, and defined amounts of pollutants in the hexane-acetone solution were used as the standards. The data represent mean values with standard deviations. Arrows indicate the time points for the metagenomic analysis.

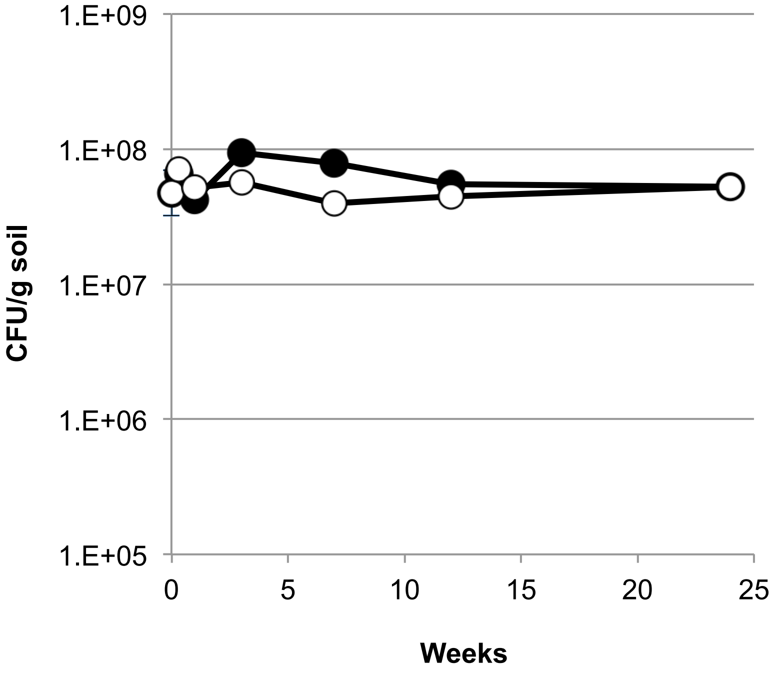

Supplementary Figure S2. Colony-forming units (CFUs) of control (open circle) and polluted (closed circle) soil-residing heterotrophic prokaryotic cells on R2A agar plates. To count the number of colony-forming heterotrophic prokaryotic cells in the soil, One-gram soil at an appropriate sampling time point was suspended in 9 ml of phosphate-buffered saline, and the suspension was diluted and spread onto Difco^{TM} R2A agar plates (Becton Dickinson, Franklin Lakes, NJ, USA) and incubated at 30°C for ten days. The data represent the mean values of triplicated measurements with standard deviations.

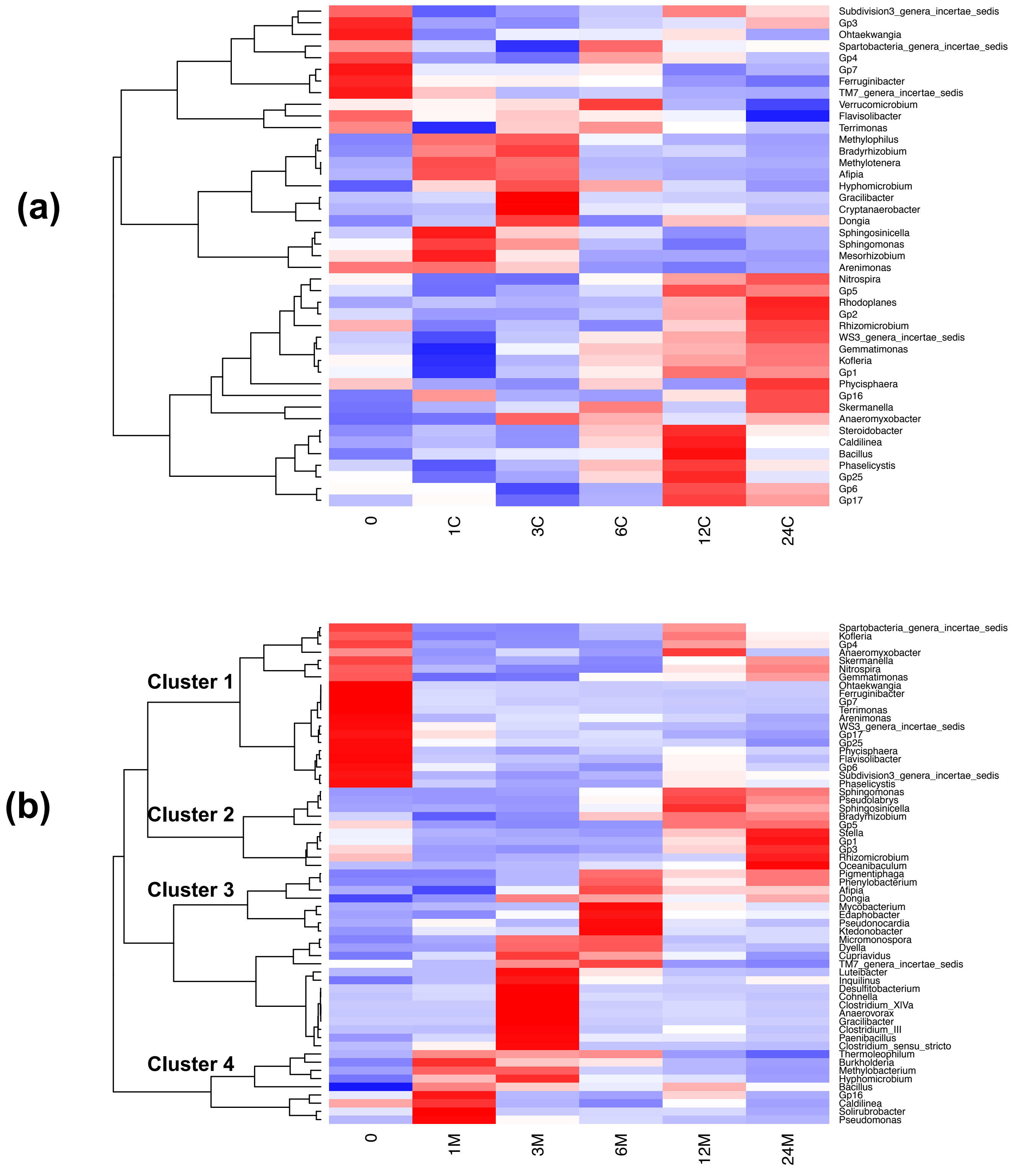

Supplementary Figure S3. Hierarchical clustering of time-course variations in abundances of the top 30 genera in control (a) and polluted (b) soils. Time-course variation patterns of relative abundances of the 30 most-abundant genera on the basis of 16S rRNA gene amplicon pyrosequencing were used for the calculation of Pearson correlation coefficients, which were subsequently subjected to hierarchical clustering.

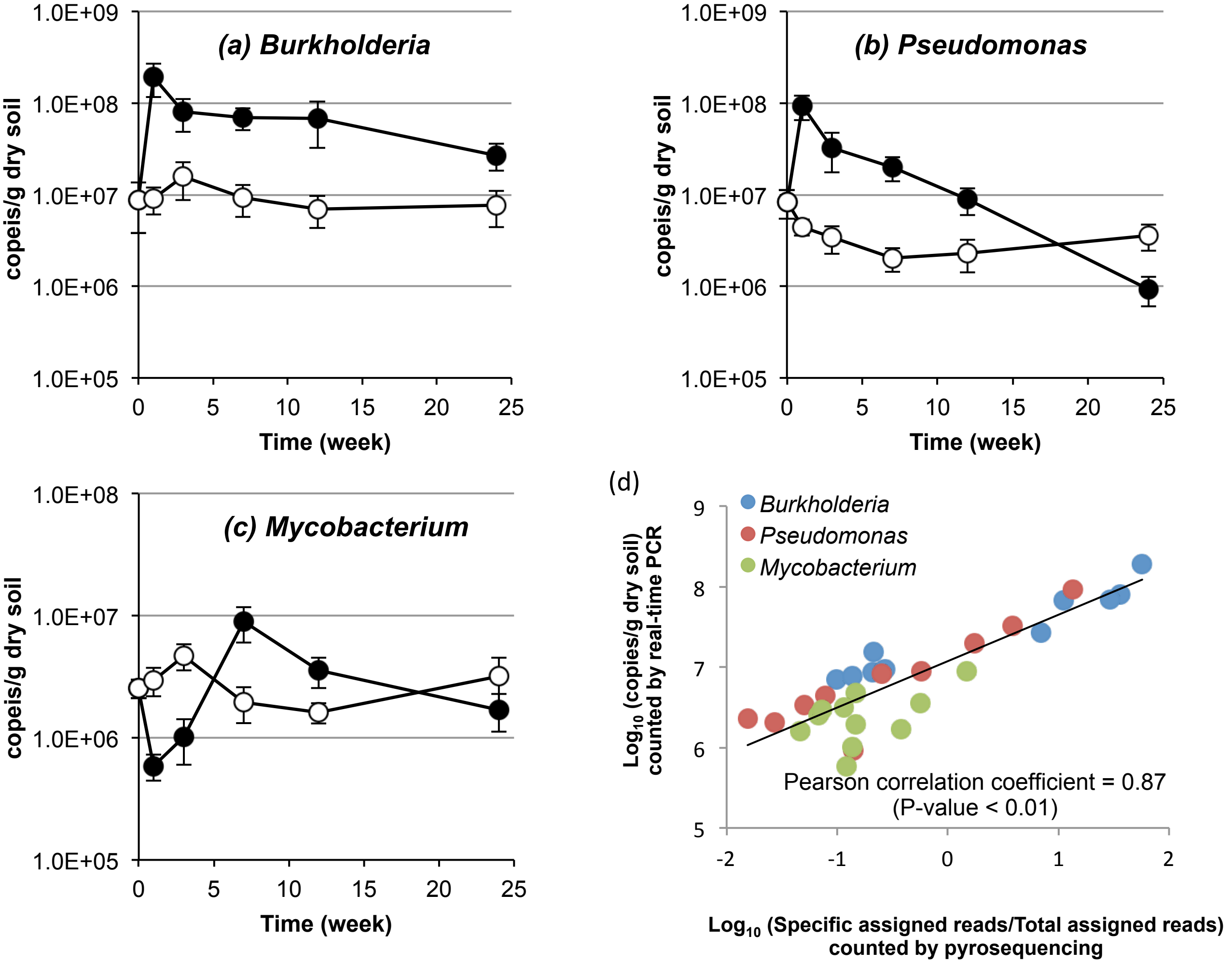

Supplementary Figure S4. Absolute copy numbers of 16S rRNA genes from *Burkholderia* (a), *Pseudomonas* (b), and *Mycobacterium* (c) in control (open circle) and polluted (closed circle) soils by quantitative PCR. The data represent the mean values of measurements using three independent DNA extracts from each soil sample. Error bars indicate the standard deviations. See below for details of the measurements. The correlation between the gene abundance quantified by quantitative PCR and that from the Roche 454 reads of 16S rRNA gene amplicons is depicted in panel **(d)**.

To quantify the absolute abundance of the three genera in the soil samples, the quantitative PCR assay developed by Park and Crowley[1] was performed using a DNA engine OpticonTM2 system (MJ Research, Waltham, MA, USA) and SYBR Premix *Ex Taq* (TAKARA BIO). A 250-mg soil sample was mixed well with the DNA solution containing 3.9 x 10^7 copies of plasmid pEGFP (Clontech, Mountain View, CA, USA)[2] as the internal standard, and the soil metagenomic DNA was extracted using the isolation kit described in Materials and methods. The recovered DNA was used as the template for the quantitative PCR with the primer set for the specific amplification of the 16S rRNA genes from the genus *Burkholderia*, *Pseudomonas*, or *Mycobacterium* as well as the primer set for amplification of the pEGFP-loaded *egfp* gene. The PCR conditions have been described elsewhere.[3-5] The copy number of the 16S rRNA genes was calculated based on the standard curve obtained by the dilution series of the DNA solution containing the 16S rRNA gene with a known concentration. The efficiency of DNA extraction from the soil was calculated as the ratio of the number of copies of the *egfp* gene detected in the recovered DNA sample to that added to the soil sample, and resulting efficiency value was used to normalize the copy number of 16S rRNA genes in the soil samples.

1. Park, J. W. and Crowley, D. E. 2005, Normalization of soil DNA extraction for accurate quantification real-time PCR and of target genes by DGGE. *Biotechniques*, **38**, 579-586.
2. Cormack, B. P., Valdivia, R. H. and Falkow, S. 1996, FACS-optimized mutants of the green fluorescent protein (GFP). *Gene*, **173**, 33-38.
3. Salles, J. F., De Souza, F. A. and van Elsas, J. D. 2002, Molecular method to assess the diversity of *Burkholderia* species in environmental samples. *Appl. Environ. Microbiol.*, **68**, 1595-1603.
4. Spilker, T., Coenye, T., Vandamme, P. and LiPuma, J. J. 2004, PCR-Based assay for differentiation of *Pseudomonas aeruginosa* from other *Pseudomonas* species recovered from cystic fibrosis patients. *J. Clin. Microbiol.*, **42**, 2074-2079.
5. Leys, N. M., Ryngaert, A., Bastiaens, L., et al. 2005, Occurrence and community composition of fast-growing *Mycobacterium* in soils contaminated with polycyclic aromatic hydrocarbons. *FEMS Microbiol. Ecol.*, **51**, 375-388.

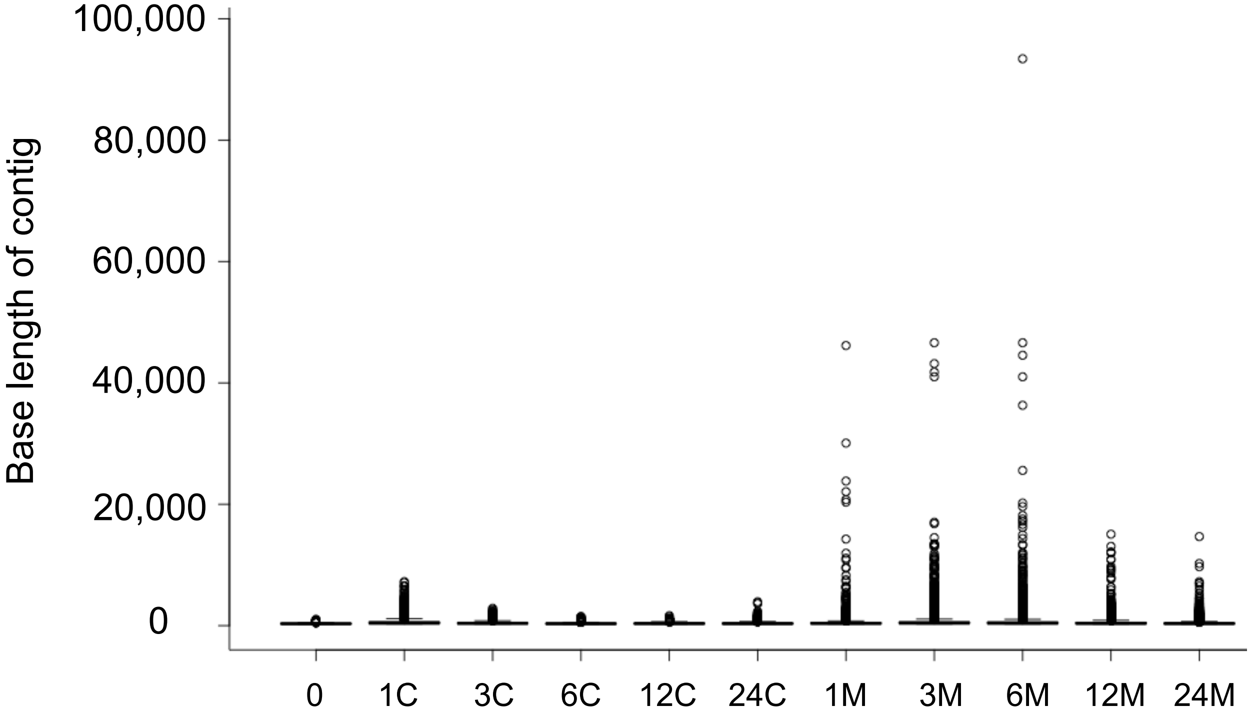

	0	1C	3C	6C	12C	24C	1M	3M	6M	12M	24M
Total reads	12,561,547	13,367,184	8,584,326	11,912,537	11,022,660	12,825,888	11,562,982	10,255,058	11,331,760	7,273,428	14,358,223
Number of contigs	724	9,232	4,396	2,467	1,469	2,735	23,131	27,654	27,155	9,792	34,887
Contig N50 length (bases)	331	683	453	347	388	393	452	621	628	520	402
The longest contig length (bases)	1,023	7,257	2,858	1,494	1,639	3,935	46,158	46,615	93,412	15,067	14,680

Supplementary Figure S5. Distribution of contig lengths in eleven metagenomic samples. Metagenomic reads from each sample were separately assembled using the IDBA-UD program.[18] The circle in the box plot represents base length of each contig. The number of reads used for the assembly, the length of contig N50, and the longest contig are indicated under the sample name.

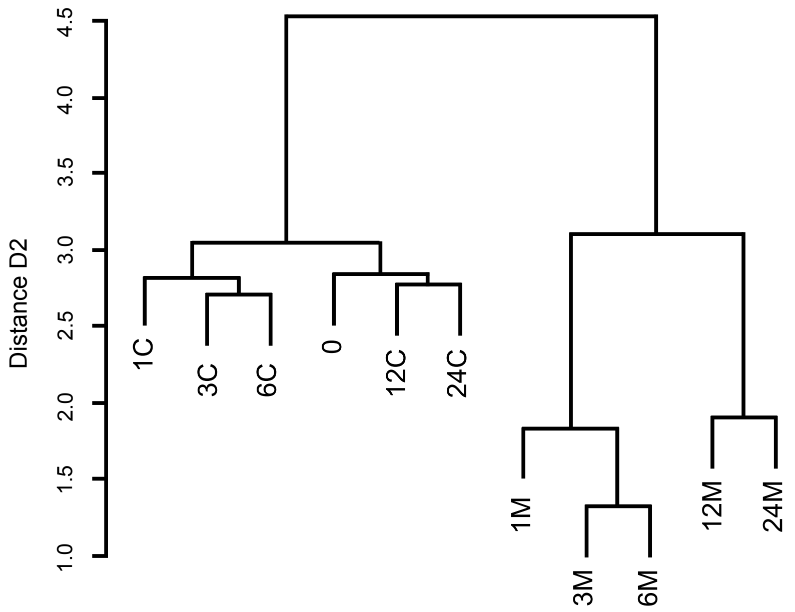

Supplementary Figure S6. Hierarchical clustering of overall gene pools among eleven metagenomic samples. All high-quality Illumina reads were used for analysis to compare the distance D2. See Materials and methods for details.

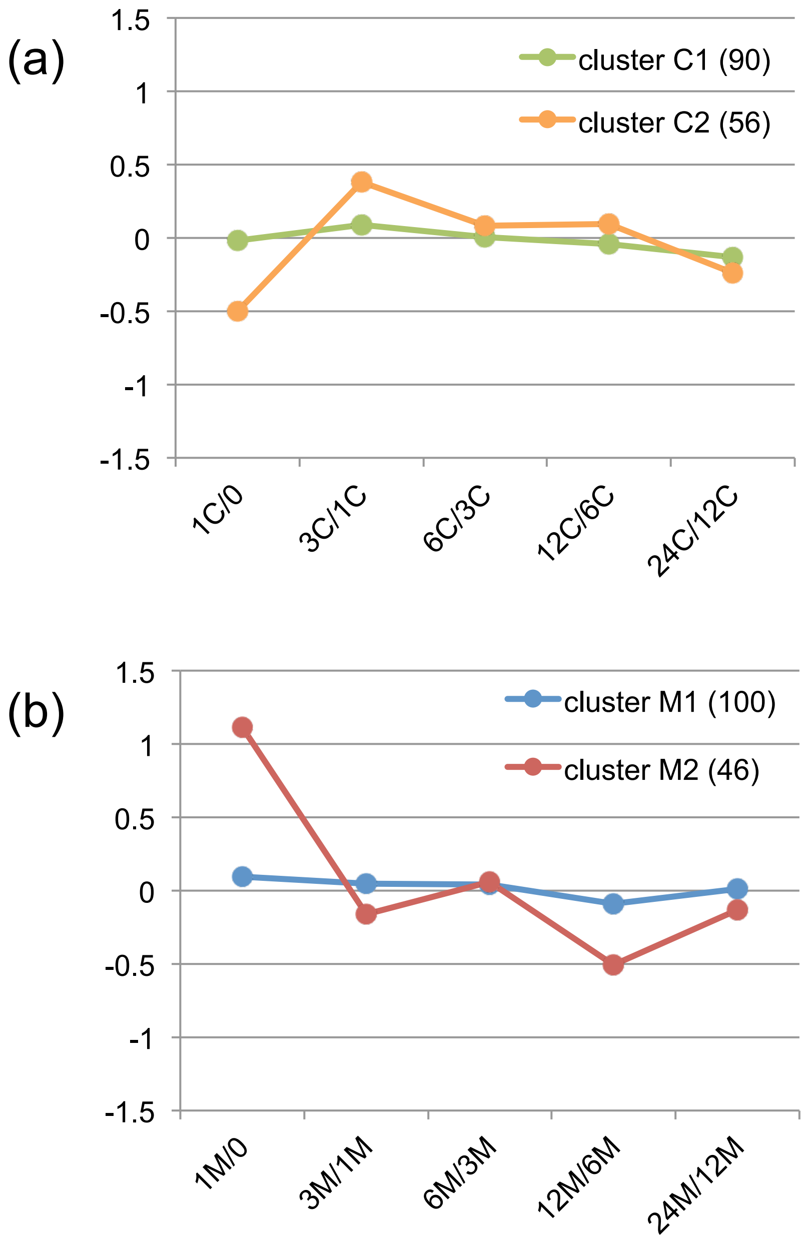

Supplementary Figure S7. Clustering of time-course variation patterns of gene abundances of KEGG pathways in metagenomic samples from control (a) and polluted (b) soils. Two clusters of variation patterns were classified by the *k*-means clustering of the variation patterns of gene abundances of KEGG pathways. The number of KEGG pathways classified into each cluster (Supplementary Tables S12 and S13) is given in parentheses. The Y-axis indicates the $\log_2$-transformed variation ratio, where the gene abundance of each pathway at one time point was divided by that at the previous time point.

(a)

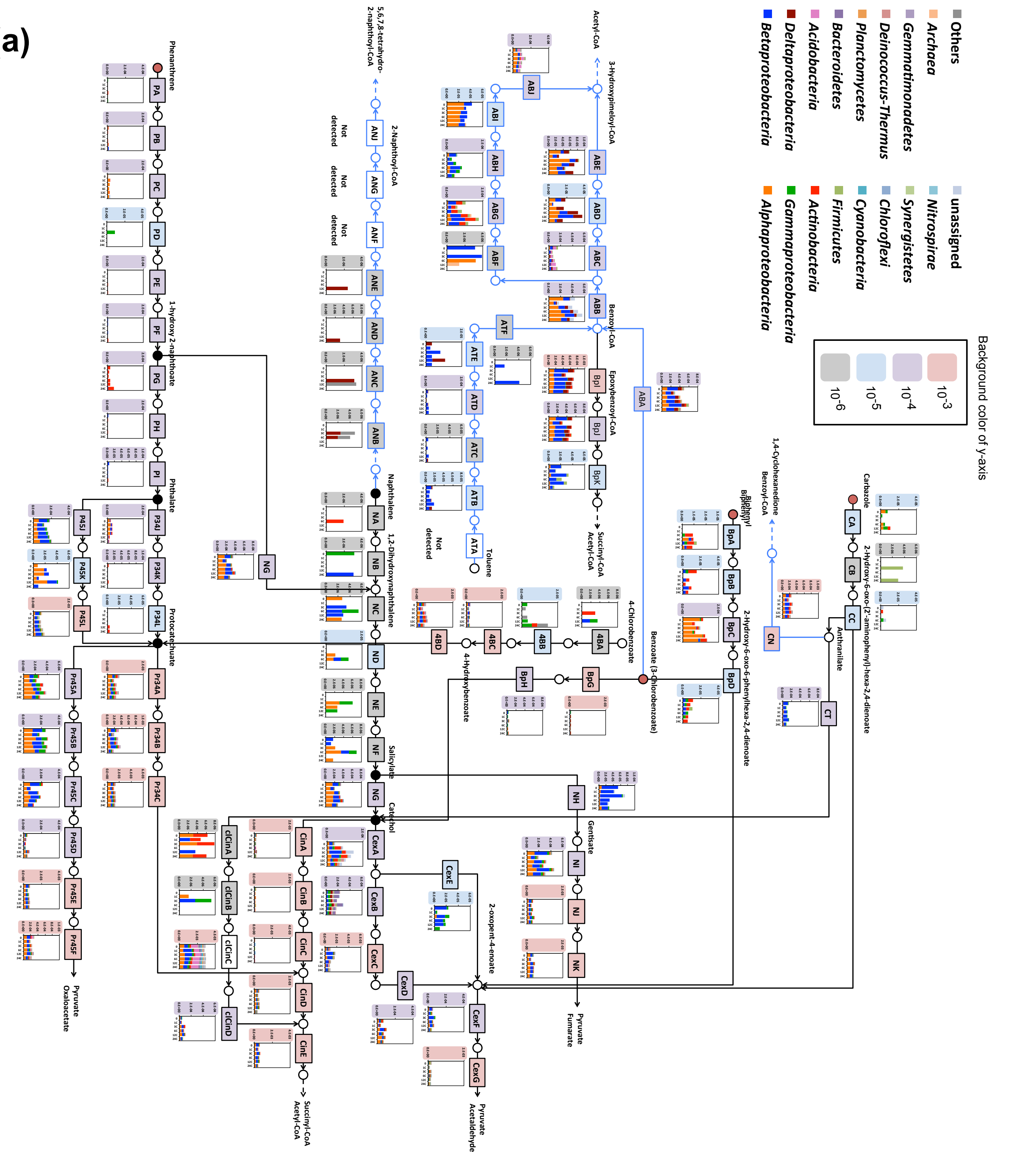

Supplementary Figure S8. Time-course changes in taxonomic compositions and abundances of genes for aerobic degradation of aromatic compounds in control (a) and polluted (b) soils. Depicted is an overall pathway map for well-known aerobic and anaerobic degradation routes of the four polluted compounds (see Fig. 4 for the simplified pathway map). Boxes located in each route indicate representative reaction steps, and several downstream steps (dashed lines) are omitted for simplicity. Abbreviations of enzymes are shown in Supplementary Table S5. The added contaminants are indicated by red nodes. The bar graph in the map indicates the abundance of the genes governing each reaction step at the phylum level (except in the cases of *Archaea* and *Proteobacteria*, for which the abundances at the domain and class levels are shown, respectively). See Materials and Methods for details of the calculation of the gene abundances. Depending on the abundances, the scales of Y-axes of graphs are conventionally categorized into four groups with the following colors: grey, 10^{-6}; blue, 10^{-5}; purple, 10^{-4}; and red, 10^{-3}.

(b)

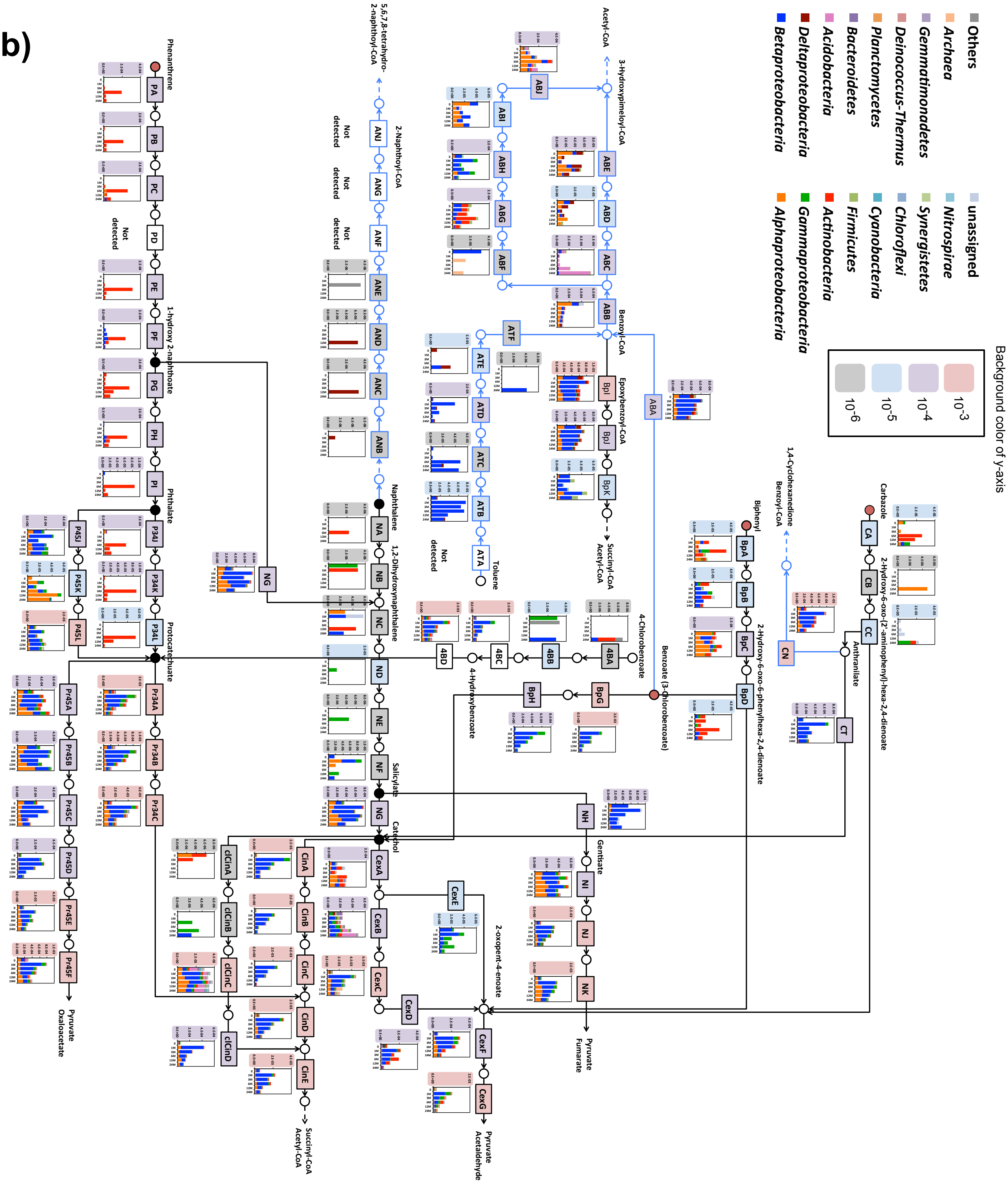

Supplementary Figure S8. continued.

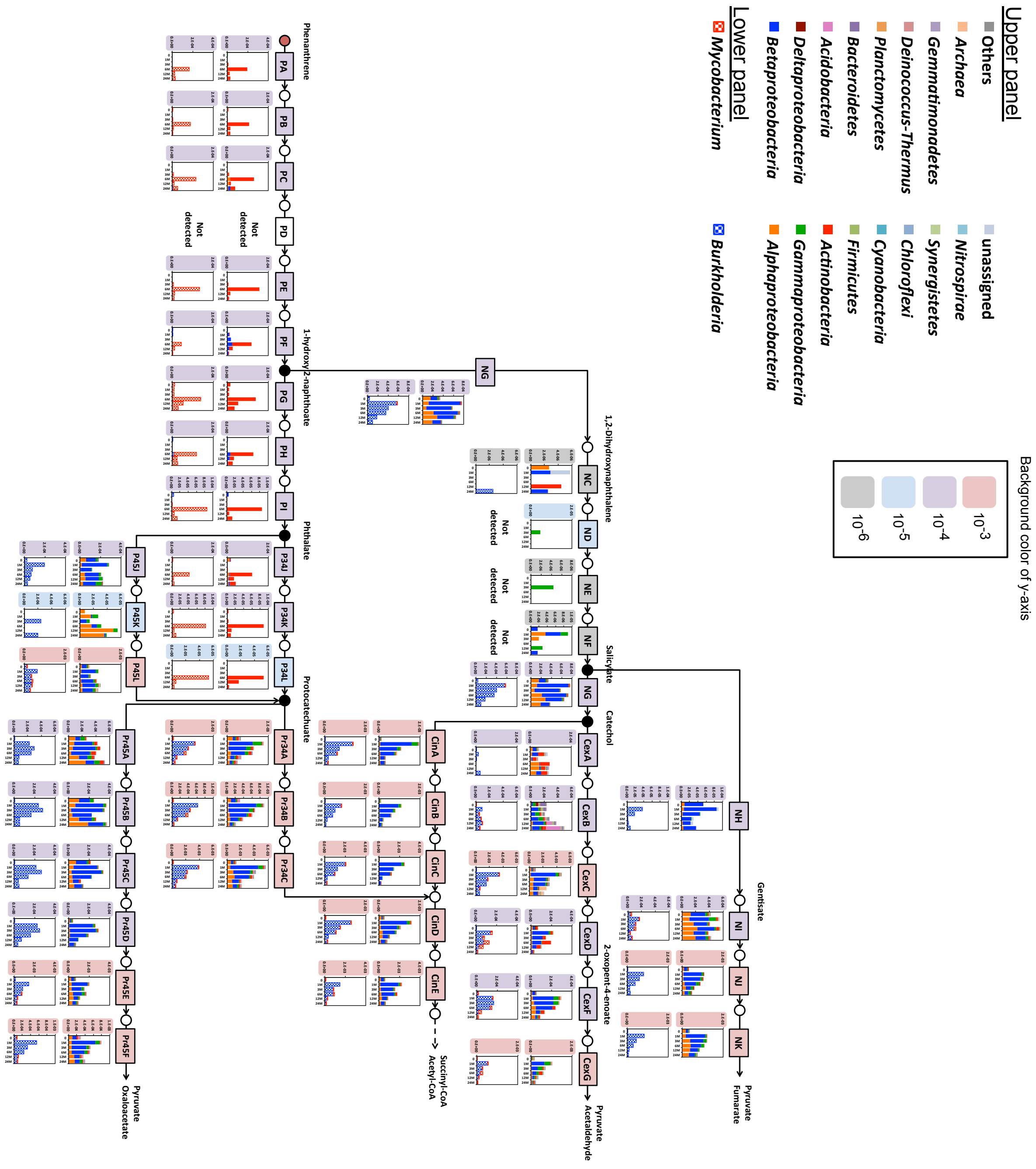

Supplementary Figure S9. Time-course changes in taxonomic compositions and abundances of genes for aerobic degradation of phenanthrene in polluted soil. Depicted is a pathway map for degradation routes of phenanthrene and its metabolites. See the legend to Supplementary Fig. S8 for the detailed explanation of this figure. The gene abundances for each reaction step in this figure are indicated by a pair of graphs. The upper graph shows the abundances at the phylum level, and the lower graph the abundances at the level limited to the genera of *Mycobacterium* and *Burkholderia*.

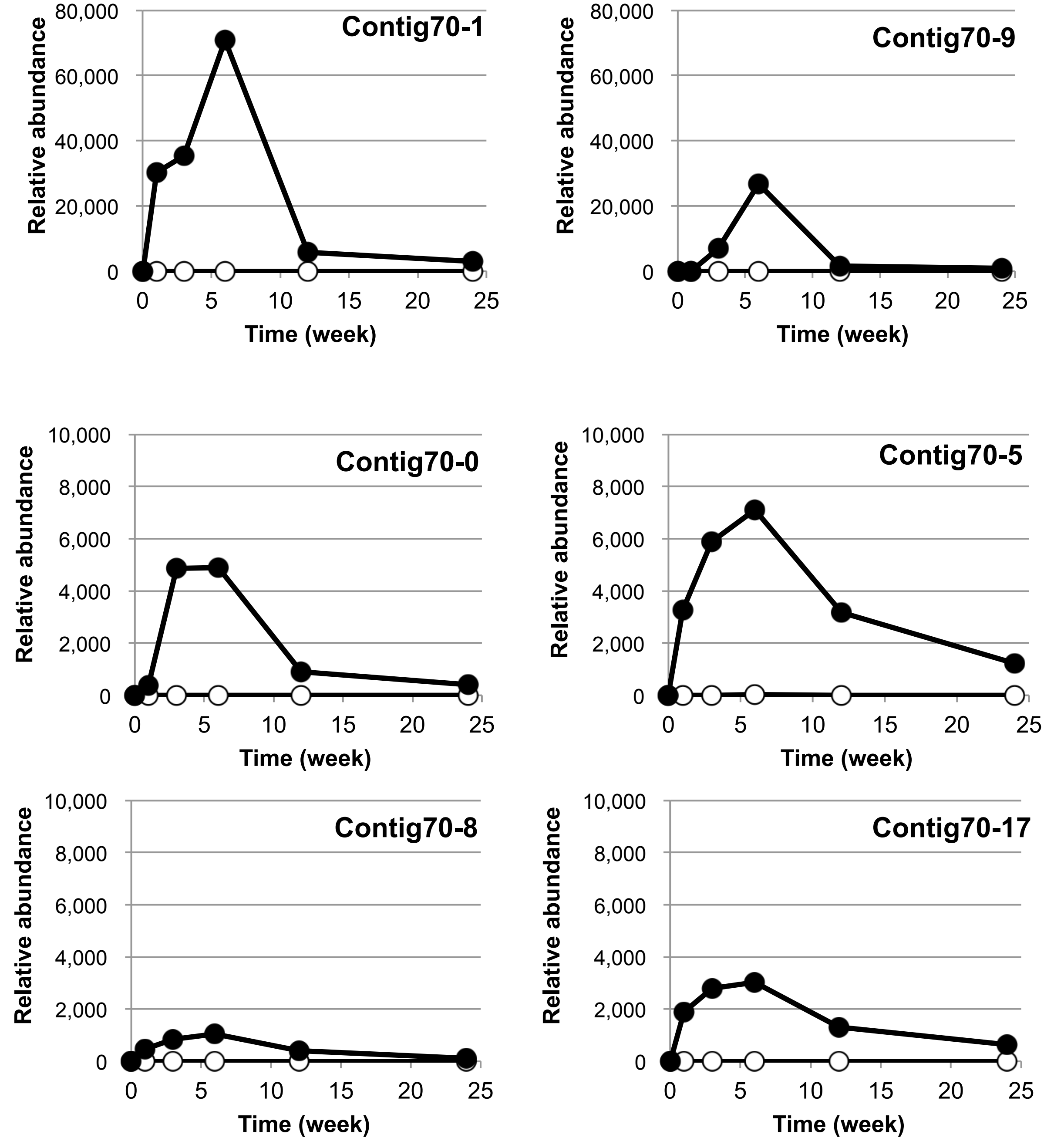

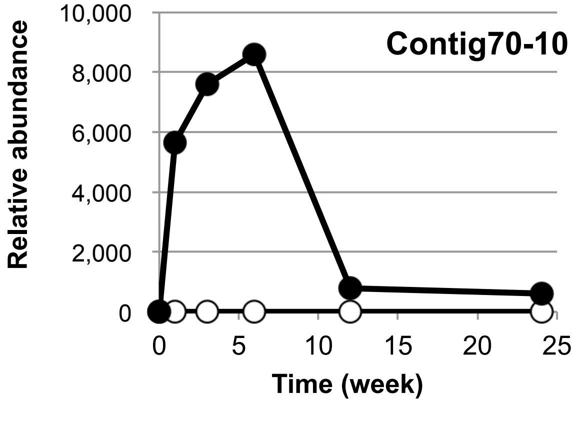

Supplementary Figure S10. Time-course changes in relative abundances of phage genome-derived sequences in soil metagenomic samples. Depicted are the relative abundances of contigs putatively derived from phage genomes in the control (open circle) and the polluted (closed circle) soil metagenomic samples (see Fig. 5b and Supplementary Fig. S11 for Contig70-3). The abundance of the phage genome-derived contig in each sample was based on the numbers of hit reads counted by the BLASTN search of metagenomic reads against the contigs. The hit numbers were normalized by the number of *gyrB*-added USCGs a in each metagenomic sample. The relative abundance values are expressed by taking the smallest value as 1.

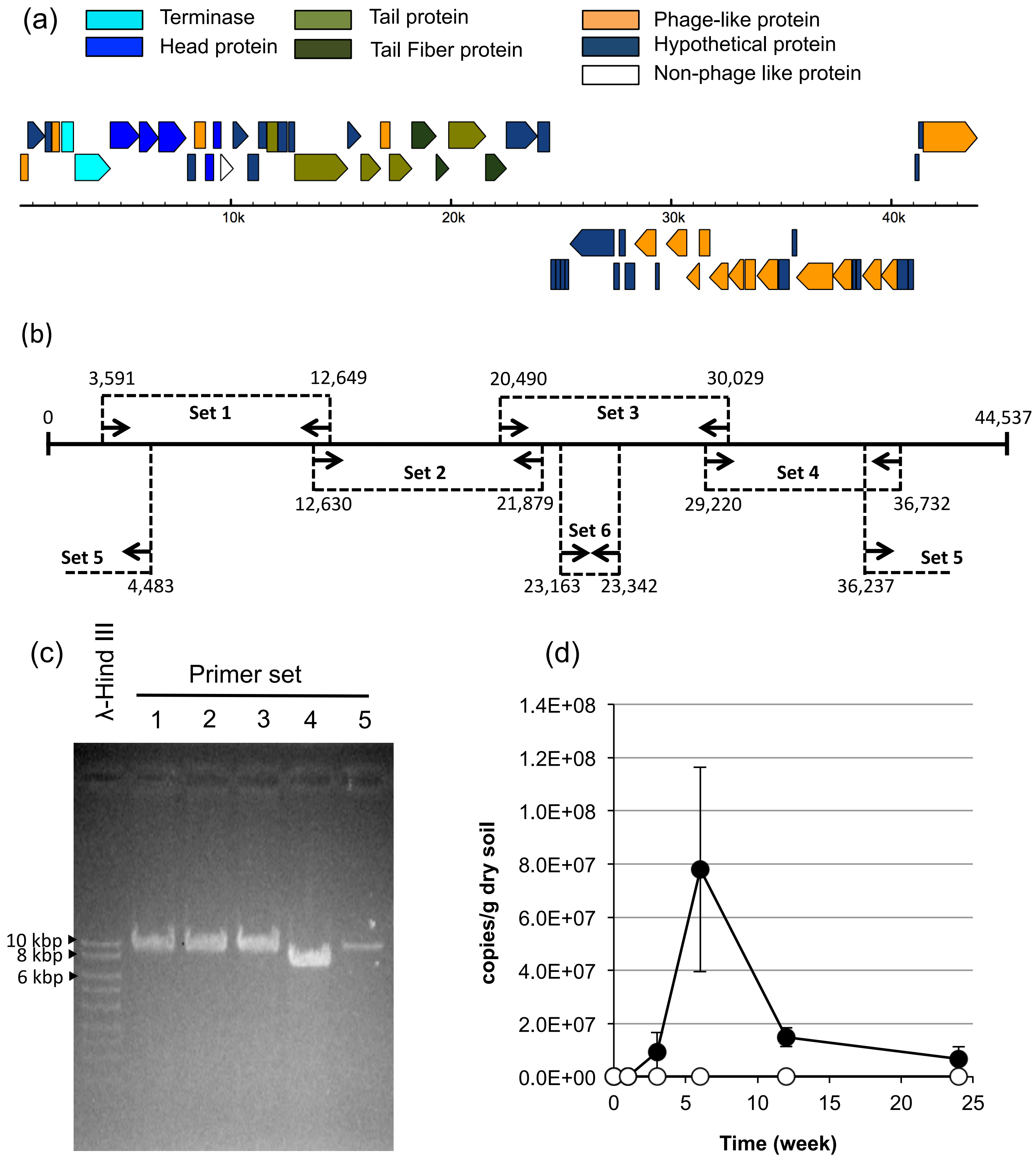

Supplementary Figure S11. PHAST analysis and PCR amplification and quantification of a 44.5-kb phage genome-derived contig (Contig70-3) in metagenomic samples.
(a) Gene organization of Contig70-3 on the basis of PHAST analysis.[33] See Table S16 for details of the PHAST analysis. **(b)** The nucleotide positions of six PCR primer sets, sets 1 to 6 (see their primer sequences in Supplementary Table S2), are depicted. The primer sets of 5 and 6 were used for PCR detection of (a) circular and/or concatenated forms of the contig and the quantitative PCR analysis, respectively. **(c)** Agarose gel electrophoresis of PCR-amplified fragments using the polluted soil metagenomic sample that was prepared at week 6. No PCR products were detected when the control soil metagenomic samples were used. **(d)** Copy number of the phage genome-derived sequence in the control (open circle) and polluted (closed circle) soil metagenomic samples. Quantitative PCR analysis was performed with three replicates.
