# Supplementary material for: Time-series metagenomic analysis reveals robustness of soil microbiome against chemical disturbance
Source: DNA Res. 2015 Oct 1;22(6):413–24. doi: 10.1093/dnares/dsv023 (PMC4675710; doi:10.1093/dnares/dsv023)
Supplement: Supplementary Data [file supp_dsv023_dsv023supp_notes.pdf]

Supplementary Notes

SN1. Preliminary survey of microbial community succession

In order to overview the taxonomic succession of soil microbial communities, the metagenomic DNA samples prepared at different time points after the pollution were used for Sanger sequencing analysis of the PCR-amplified 16S rRNA genes and PCR-denaturing gradient gel electrophoresis (PCR-DGGE) analysis of the 16S rRNA genes. The metagenomic DNA samples prepared from (i) the soil sample at the time just before the pollution and (ii) the control and polluted soil samples at weeks 6, 12, and 22 after the pollution were used as the templates to construct a small-scale library of these genes. Parts of prokaryotic16S rRNA genes between the V3 and V5 regions were amplified by PCR using a primer set of 338F (5'-ACTCCTACGGGAGGCAGCAG-3') and 907R (5'-CCGTCAATTCCTTTRAGTTT-3')[1,2] (the references cited in this file are listed in below), and KOD DNA polymerase (TOYOBO, Osaka, Japan). The PCR reaction was carried out as follows: 94°C for 2 min, followed by 40 cycles of 94°C for 30 sec, 52°C for 30 sec, and 68°C for 30 sec. The amplicons were cloned into an *Hin*cII site of plasmid pHSG299,[3] and the resulting recombinant plasmids were introduced into *Escherichia coli* DH5α by transformation.[4] Approximately 80 amplicons per soil sample were sequenced by an Applied Biosystems 3730xl DNA Analyzer at the Dragon Genomics Center of TAKARA BIO (Mie, Japan), and were subjected to the taxonomic assignment using the RDP classifier.[5] Our Sanger sequencing of such libraries indicated that the community compositions in the control soil apparently remained unchanged except for a transient increase of the genus *Methylophilus* in the class *Betaproteobacteria* at week 6, and that the microbial community compositions in the polluted soil exhibited a drastic increase at week 6 of the phylum *Proteobacteria*, especially the genus *Dyella* in the class *Gammaproteobacteria*, the genera *Dongia* and *Phenylobacterium* in the class *Alphaproteobacteria*, and *Burkholderia* in the class *Betaproteobacteria* (Fig. SN1ab). Hierarchical clustering of these taxonomic compositions revealed the drastic changes in the dominant microbial members by the addition of pollutants (Fig. SN1c).

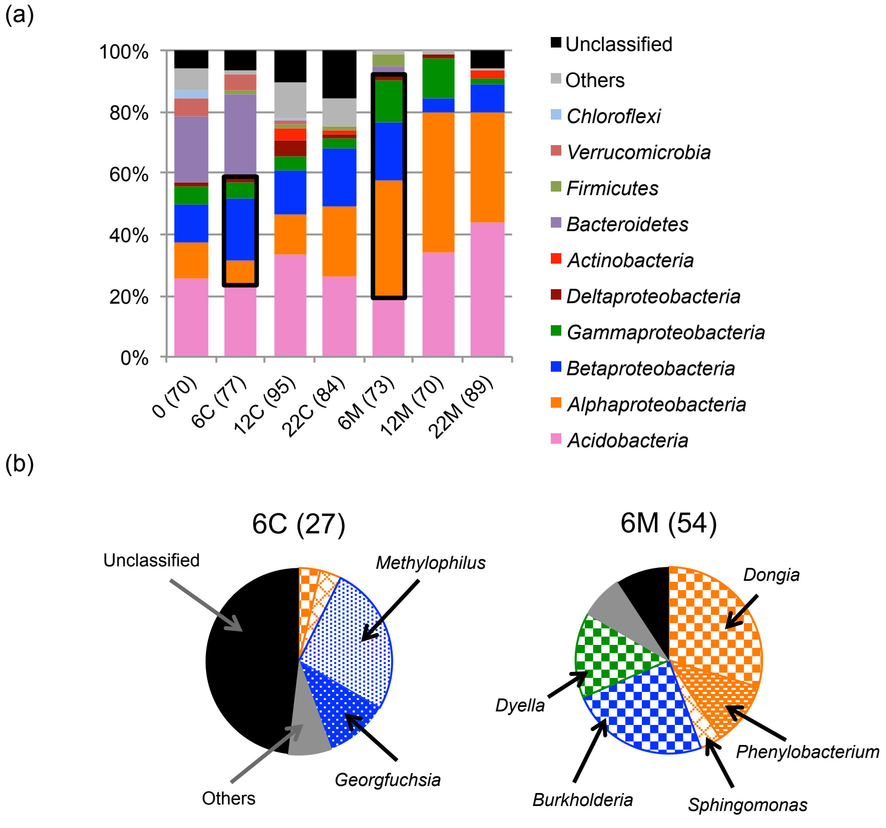

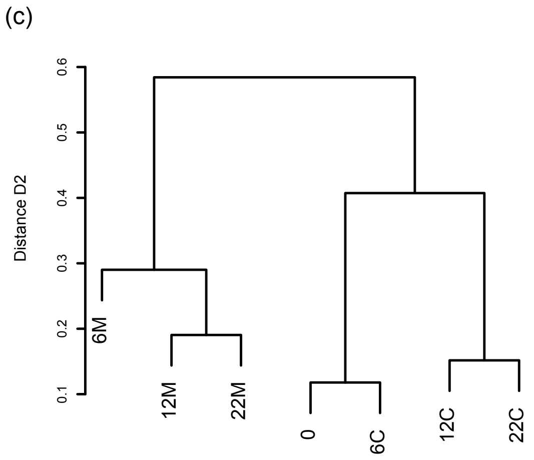

Figure SN1. Phylum-level analysis of 16S rRNA gene (V3-V5 region) libraries. (a) Approximately eighty pHSG299-inserted amplicon clones were Sanger-sequenced and taxonomically assigned at the phylum level (except in the case of *Proteobacteria* at the class level) by using the RDP classifier. The 16S rRNA gene amplicon library was constructed from the metagenomic DNA prepared from control (C) or polluted (M) soil at three time points. The numerals before C and M are the weeks after the pollution of soil samples. The number of clones used for the taxonomic annotation is indicated in parentheses. **(b)** Genus-level taxonomic assignments of the clones in *Proteobacteria* at week 6 [black boxes in panel **(a)**]. **(c)** Hierarchical clustering of the phylum-level taxonomic compositions, by comparison of the Euclidean distances.

Preliminary survey of the microbial community succession was also examined by PCR-DGGE analysis according to Morimoto et al.[6] with some modifications. Parts of the 16S rRNA genes were amplified by PCR using the metagenomic DNA as the template, a primer set of 338F with GC clump (5’-CGCCCGCCGCGCGCGGCGGGCGGGGCGGGGGCACGGGGGG-3’)[7] and 907R, and *Ex Taq* polymerase (TAKARA BIO, Ohtsu, Japan). The PCR reaction condition was as follows: 95°C for 2 min, followed by 30 cycles of 95°C for 30 sec, 55°C for 30 sec, and 72°C for 30 sec, and finally at 72°C for 7 min. Each 200-ng portion of amplicons from each metagenomic DNA sample was loaded into each lane

well of the 6% polyacrylamide gel with the denaturing gradient of 45-70%, and electrophoresis was performed using a D-code universal mutation detection system (BIO-RAD Laboratories, Hercules, CA, USA) according to the manufacturer's instruction. The electrophoresis band patterns were visualized by staining the gel with SYBR Green. DGGE patterns of 16S rRNA genes (Fig. SN2) showed that the addition of pollutants resulted in (i) the immediate appearance of several intense bands, a few of which were assigned to be of *Burkholderia* origin by the Sanger sequencing and, and (ii) the fluctuation of the community compositions at least during the 24-week incubation period.

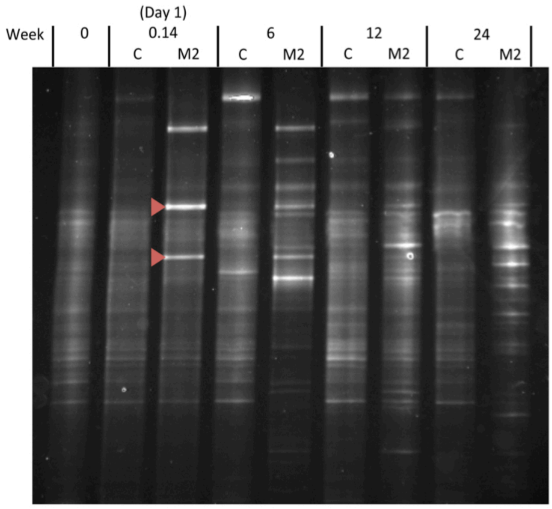

Figure SN2. DGGE profiles of PCR-amplified V3-V5 regions of 16S rRNA genes in control (C) and polluted (M) soil samples. The intense bands with a red triangle are the 16S rRNA gene amplicons derived from *Burkholderia*.

To investigate the time-course changes in microbial potentials for degradation of the pollutants in the soil samples, the relative abundances of genes for catalytic subunits of various types of well-known dioxygenases for the degradation of aromatic compounds were investigated by quantitative PCR assays using the metagenomic DNA as the template and the primer sets for the amplification of many well-characterized oxygenase genes (Table SN1) using a DNA engine OpticonTM2 system (MJ Research, Waltham, MA, USA) and SYBR Premix *Ex Taq* (TAKARA BIO).

Table SN1. Primer sets used for preliminary survey of oxygenase genes for degradation of aromatic compounds.

Target gene function	Primer set	References
PAH dioxygenase *(pahAc, nahAc, nagAc*, Gram-negative)	PAH-RHDα GN F / PAH-RHDα GN R	8
PAH dioxygenase (*nidA3, narAa, pdoA*, Gram-positive) *	PAH-RHDα GP F / PAH-RHDα GP R	8
PAH dioxygenase (*nah*-like)	pPAH-F / pPAH-NR700	9
PAH dioxygenase (*nah*-like)	NAH-F / NAH-R	10
PAH dioxygenase (*nah*-like)	nahAcfor / nahAcrev	11
PAH dioxygenase (*phn*-like)	P8073 / P9047	11
PAH dioxygenase (*dxn*-like)	AJ025 / AJ026	12
PAH dioxygenase (*pdoA* and *nidA*-like, Gram-positive) *	pdo1-f / pdo1-r	13
PAH dioxygenase (*nidA*)	Nid-for / Nid-rev1	14
Biphenyl dioxygenase	BPH1-F / BPH1-R	10
Biphenyl dioxygenase	BPH3-F / BPH3-R	10
Biphenyl dioxygenase	bphAf668-3 / bphAr1153-2	15
Carbazole dioxygenase (*carAa*)	carAa F / carAa R	16
Catechol dioxygenase	XYLE1-F / XYLE1-R	17
Toluene dioxygenase	TOD-F / TOD-R	10
Xylene monooxygenase	TOL-F / TOL-R	10
Toluene monooxygenase	RMO-F / RMO-R	10
Benzoate 1,2-dioxygenase (*benA*) *	BAF1 / BAR1	6

Several primer sets different in primer annealing positions were used to amplify the genes for naphthalene, biphenyl, and phenanthrene dioxygenases. PCR amplification was carried out using the soil metagenomic samples prepared at weeks 5, 6, 8, and 11 after the pollution. PCR products were successfully obtained only when the primer sets with asterisks were used. The primer sequences and PCR conditions were those described in the references.

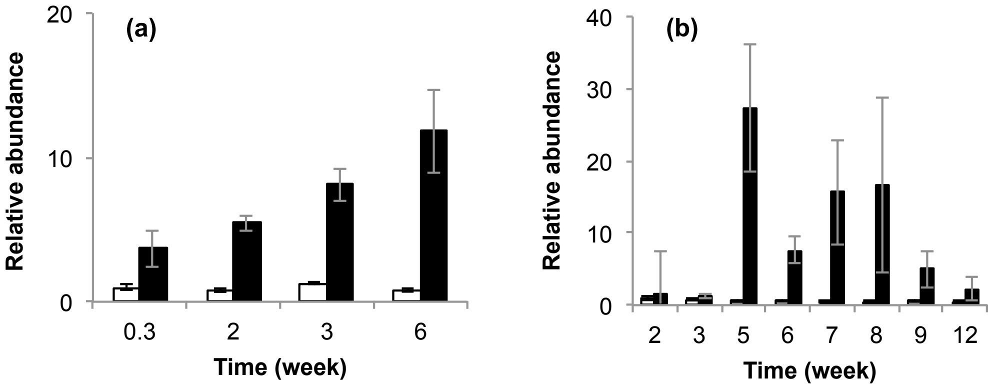

Figure SN3. Relative abundance of *benA* (a) and *pdoA*-like (b) genes in control (white) and polluted (black) soils by quantitative PCR analysis. Values are expressed by taking the value of the control soil at the initial time point (week 0) as 1. The data represent the mean values of triplicated measurements with standard deviations.

This investigation showed that the gram-negative bacterial types of genes for benzoate dioxygenases, which can also act as the first enzymes for aerobic 3-chlorobenzoate (3CB) degradation,[18] and the gram-positive bacterial types of genes for PAH dioxygenases, the first enzymes for the aerobic degradation of pyrene and phenanthrene, were abundant in the polluted soil (Fig. SN3). On the other hand, no PCR products were detected when other primer sets for the amplification of genes for well-known biphenyl dioxygenases and carbazole dioxygenases (Table SN1) were used.

SN2. Isolation and draft genome sequencing of bacterial strains from polluted soil

In order to obtain genome information on the indigenous bacterial strains residing in the polluted soil, bacterial strains from several polluted soil samples were isolated, and their draft genomes were sequenced. Since our amplicon sequencing of 16S rRNA genes from polluted soil indicated that *Burkholderia* was the most proliferated genus (see Results and Discussion), serial dilutions of the soil suspension in an appropriate volume of phosphate-buffered saline were plated onto PCAT (*Pseudomonas cepacia* azelaic acid tryptamine) agar, a semi-selection medium for *Burkholderia* strains.[19] Single colonies formed after incubation at 30°C for ten days were repeatedly streaked on the same medium for purification, and their taxonomic assignment was confirmed by PCR amplification of the *Burkholderia*-specific 16S rRNA gene (see below). Finally, two *Burkholderia* strains, E7m39 and E7m48, were obtained from the soil samples at week 1 after the pollution, and one such strain, E168m22, at week 24. To isolate the aromatic compound-degrading bacterial strains, three gram-portion of a polluted soil sample at week 15 was transferred to 50 ml of 1/10-strength W (1/10W) minimal liquid medium[20] supplemented with 0.1 mg/ml of phenanthrene, biphenyl, or carbazole as a sole source of carbon and energy. After incubation at 30°C for two weeks, only the phenanthrene-containing culture exhibited its significant degradation activity. The bacterial strains capable of degrading this compound were enriched by repeated subcultures in the same liquid medium, and purified by spreading onto a phenanthrene-containing 1/10 W agar plate. One such strain designated *Mycobacterium* sp. EPa45 indeed degraded phenanthrene to TCA compounds, and our gas chromatography-mass spectrometer analysis showed that EPa45 was able to completely degrade 0.2 mM phenanthrene within seven days of incubation in the minimal liquid medium.

Genomic DNA of the four strains thus isolated were prepared using the nexttec™ Genomic DNA Isolation Kit from Bacteria (Nexttec GmbH, Leverkusen, Germany), mechanically sheared to obtain libraries of approximately 8 kb in length, and subjected to mate-pair pyrosequencing using the Roche 454 GS FLX platform and its standard protocol. The genome sequences assembled by Newbler version 2.8 with default parameters were included in our genome sequence dataset for the assignment of Illumina reads of soil metagenomes.[21]

References

1. Ovreas, L., Forney, L., Daae, F. L. and Torsvik, V. 1997, Distribution of bacterioplankton in meromictic Lake Saelenvannet, as determined by denaturing gradient gel electrophoresis of PCR-amplified gene fragments coding for 16S rRNA. *Appl. Environ. Microbiol.*, **63**, 3367-3373.
2. Lane, D. J., Pace, B., Olsen, G. J., Stahl, D. A., Sogin, M. L. and Pace, N. R. 1985, Rapid determination of 16S ribosomal RNA sequences for phylogenetic analyses. *Proc. Natl. Acad. Sci. U. S. A.*, **82**, 6955-6959.
3. Takeshita, S., Sato, M., Toba, M., Masahashi, W. and Hashimoto-Gotoh, T. 1987, High-copy-number and low-copy-number plasmid vectors for *lacZ* alpha-complementation and chloramphenicol- or kanamycin-resistance selection. *Gene*, **61**, 63-74.
4. Sambrook, J. and W. Russell, D. W. 2001, *Molecular Cloning : A Laboratory Manual*. Cold Spring Harbor Laboratory Press: New York, USA.
5. Wang, Q., Garrity, G. M., Tiedje, J. M. and Cole, J. R. 2007, Naive Bayesian classifier for rapid assignment of rRNA sequences into the new bacterial taxonomy. *Appl. Environ. Microbiol.*, **73**, 5261-5267.
6. Morimoto, S., Togami, K., Ogawa, N., Hasebe, A. and Fujii, T. 2005, Analysis of a bacterial community in 3-chlorobenzoate-contaminated soil by PCR-DGGE targeting the 16S rRNA gene and benzoate 1,2-dioxygenase gene (*benA*). *Microbes Environ.*, **20**, 151-159.
7. Muyzer, G., Dewaal, E. C. and Uitterlinden, A. G. 1993, Profiling of complex microbial populations by denaturing gradient gel-electrophoresis analysis of polymerase chain reaction-amplified genes coding for 16S rRNA. *Appl. Environ. Microbiol.*, **59**, 695-700.
8. Cebron, A., Norini, M. P., Beguiristain, T. and Leyval, C. 2008, Real-time PCR quantification of PAH-ring hydroxylating dioxygenase (PAH-RHDα) genes from gram positive and gram negative bacteria in soil and sediment samples. *J. Microbiol. Methods*, **73**, 148-159.
9. Hedlund, B. P., Geiselbrecht, A. D., Bair, T. J. and Staley, J. T. 1999, Polycyclic aromatic hydrocarbon degradation by a new marine bacterium, *Neptunomonas naphthovorans* gen. nov., sp. nov. *Appl. Environ. Microbiol.*, **65**, 251-259.
10. Baldwin, B. R., Nakatsu, C. H. and Nies, L. 2003, Detection and enumeration of aromatic oxygenase genes by multiplex and real-time PCR. *Appl. Environ. Microbiol.*, **69**, 3350-3358.
11. Lloyd-Jones, G., Laurie, A. D., Hunter, D. W. F. and Fraser, R. 1999, Analysis of catabolic genes for naphthalene and phenanthrene degradation in contaminated New Zealand soils. *FEMS Microbiol. Ecol.*, **29**, 69-79.
12. Armengaud, J., Happe, B. and Timmis, K. N. 1998, Genetic analysis of dioxin dioxygenase of *Sphingomonas* sp. Strain RW1: Catabolic genes dispersed on the genome. *J. Bacteriol.*, **180**, 3954-3966.
13. Johnsen, A. R., de Lipthay, J. R., Sorensen, S. J., et al. 2006, Microbial degradation of street dust polycyclic aromatic hydrocarbons in microcosms simulating diffuse pollution of urban soil. *Environ. Microbiol.*, **8**, 535-545.
14. Zhou, H. W., Guo, C. L., Wong, Y. S. and Tam, N. F. 2006, Genetic diversity of dioxygenase genes in polycyclic aromatic hydrocarbon-degrading bacteria isolated from mangrove sediments. *FEMS Microbiol. Lett.*, **262**, 148-157.
15. Witzig, R., Junca, H., Hecht, H. J. and Pieper, D. H. 2006, Assessment of toluene/biphenyl dioxygenase gene diversity in benzene-polluted soils: Links between benzene biodegradation and genes similar to those encoding isopropylbenzene dioxygenases. *Appl. Environ. Microbiol.*, **72**, 3504-3514.
16. Habe, H., Ashikawa, Y., Saiki, Y., Yoshida, T., Nojiri, H. and Omori, T. 2002, *Sphingomonas* sp strain KA1, carrying a carbazole dioxygenase gene homologue, degrades chlorinated dibenzo-p-dioxins in soil. *FEMS Microbiol. Lett.*, **211**, 43-49.
17. Hendrickx, B., Junca, H., Vosahlova, J., et al. 2006, Alternative primer sets for PCR detection of genotypes involved in bacterial aerobic BTEX degradation: Distribution of the genes in BTEX degrading isolates and in subsurface soils of a BTEX contaminated industrial site. *J. Microbiol. Methods*, **64**, 250-265.
18. Ogawa, N., Miyashita, K. and Chakrabarty, A. M. 2003, Microbial genes and enzymes in the degradation of chlorinated compounds. *Chem. Rec.*, **3**, 158-171.
19. Pallud, C., Viallard, V., Balandreau, J., Normand, P. and Grundmann, G. 2001, Combined use of a specific probe and

PCAT medium to study *Burkholderia* in soil. *J. Microbiol. Methods*, **47**, 25-34.

20. Ito, M., Prokop, Z., Klvana, M., et al. 2007, Degradation of beta-hexachlorocyclohexane by haloalkane dehalogenase LinB from gamma-hexachlorocyclohexane-utilizing bacterium *Sphingobium* sp. MI1205. *Arch. Microbiol.*, **188**, 313-325.
21. Kato, H., Ogawa, N., Ohtsubo, Y., et al. 2015, Complete genome sequence of a phenanthrene degrader, *Mycobacterium* sp. strain EPa45 (NBRC 110737), isolated from a phenanthrene-degrading consortium. *Genome Announc.*, **3**, e00782-15.
